# RALB GTPase: a critical regulator of DR5 expression and TRAIL sensitivity in *KRAS* mutant colorectal cancer

**DOI:** 10.1038/s41419-020-03131-3

**Published:** 2020-10-29

**Authors:** Hajrah Khawaja, Andrew Campbell, Jamie Z. Roberts, Arman Javadi, Paul O’Reilly, Darragh McArt, Wendy L. Allen, Joanna Majkut, Markus Rehm, Alberto Bardelli, Federica Di Nicolantonio, Christopher J. Scott, Richard Kennedy, Nicolas Vitale, Timothy Harrison, Owen J. Sansom, Daniel B. Longley, Emma Evergren, Sandra Van Schaeybroeck

**Affiliations:** 1 Drug Resistance Group, Patrick G. Johnston Centre for Cancer Research, School of Medicine, Dentistry and Biomedical Science, Queen’s University Belfast, 97 Lisburn Road, Belfast, BT9 7AE UK; 2 Cancer Research UK Beatson Institute, Switchback Road, Bearsden, Glasgow, G61 1BD UK; 3 Institute of Cell Biology and Immunology, University of Stuttgart, Allmandring 31, D-70569 Stuttgart, Germany; 4 Department of Oncology, University of Torino, Candiolo, TO 10060 Italy; 5 Candiolo Cancer Institute, FPO-IRCCS, Candiolo, TO 10060 Italy; 6 Centre National de la Recherche Scientifique, Université de Strasbourg, Institut des Neurosciences Cellulaires et Intégratives, F-67000 Strasbourg, France; 7 Institute of Cancer Sciences, University of Glasgow, Garscube Estate, Switchback Road, Glasgow, G61 1QH UK

**Keywords:** Colon cancer, Oncogenes, Apoptosis

## Abstract

*RAS* mutant (MT) metastatic colorectal cancer (mCRC) is resistant to MEK1/2 inhibition and remains a difficult-to-treat group. Therefore, there is an unmet need for novel treatment options for *RAS*MT mCRC. RALA and RALB GTPases function downstream of RAS and have been found to be key regulators of several cell functions implicated in KRAS-driven tumorigenesis. However, their role as regulators of the apoptotic machinery remains to be elucidated. Here, we found that inhibition of RALB expression, but not RALA, resulted in Caspase-8-dependent cell death in *KRAS*MT CRC cells, which was not further increased following MEK1/2 inhibition. Proteomic analysis and mechanistic studies revealed that RALB depletion induced a marked upregulation of the pro-apoptotic cell surface TRAIL Death Receptor 5 (DR5) (also known as TRAIL-R2), primarily through modulating DR5 protein lysosomal degradation. Moreover, DR5 knockdown or knockout attenuated siRALB-induced apoptosis, confirming the role of the extrinsic apoptotic pathway as a regulator of siRALB-induced cell death. Importantly, TRAIL treatment resulted in the association of RALB with the death-inducing signalling complex (DISC) and targeting RALB using pharmacologic inhibition or RNAi approaches triggered a potent increase in TRAIL-induced cell death in *KRAS*MT CRC cells. Significantly, high *RALB* mRNA levels were found in the poor prognostic Colorectal Cancer Intrinsic Subtypes (CRIS)-B CRC subgroup. Collectively, this study provides to our knowledge the first evidence for a role for RALB in apoptotic priming and suggests that RALB inhibition may be a promising strategy to improve response to TRAIL treatment in poor prognostic *RAS*MT CRIS-B CRC.

## Introduction

Mutations in *RAS* (*KRAS* and *NRAS*) in exon 2 (codon 12, 13), 3 (codon 59, 61) and 4 (codon 117, 146) occur in 55–60% of colorectal cancer (CRC) patients^1^ and have been associated with poor survival^2^. Activating mutations in *RAS* lead to aberrant activation of RAS downstream effector signalling either by increasing its intrinsic GDP/GTP exchange rate or by reducing the rate of its intrinsic and GTP-ase activating protein (GAP)-induced hydrolysis^3^. RAS has proven difficult to target and inhibition of its downstream effectors MEK1/2 and AKT/PI3K has been shown to be ineffective in *RAS* mutant (MT) CRC^4,5^.

Like RAS, the RAS-like (RAL) small GTPases RALA and RALB are activated by RAL-selective guanine nucleotide exchange factors (RAL-GEFs), such as the RAL guanine nucleotide dissociation stimulator (RALGDS), and are inactivated by GTPase activating proteins (RALGAPs). RALGDS couples RAS to the RAL proteins, stimulating the GDP/GTP exchange of RAL. In their active GTP-bound state, RALA and RALB can interact with a range of functionally different effectors, including RALBP1, EXO84 and SEC5, two distinct subunits of the hetero-octomeric exocyst complex, as well as with ZO-1-associated nucleic acid-binding protein (ZONAB)^6^. Previous studies have established the requirement for RAL-GEFs for the transformation of human epithelial cells^7,8^. Using a KRAS-driven murine lung carcinoma model, other investigators have shown that RALA or RALB activity is required for tumour growth^9^. RALA and RALB have also been shown to have opposing and/or distinct roles in migration and anchorage-independent growth in bladder, prostate and pancreatic cancer, respectively^10–12^.

In this study, we report a novel role for RALB in regulating Death Receptor 5 (DR5) protein levels and cell death in *KRAS*MT CRC. Cellular and mechanistic studies indicate that RALB associates with DR5 and the death-inducing signalling complex (DISC) in a ligand-dependent manner, and that inhibition of RALB combined with DR5 agonists can be a novel treatment strategy for poor prognostic Colorectal Cancer Intrinsic Subtype (CRIS)-B^13^ *KRAS*MT CRC.

## Materials and methods

### Materials

AZD6244, recombinant human (rh)TRAIL and z-VAD-FMK were purchased from AstraZeneca (Macclesfield, UK), Calbiochem (Hertfordshire, UK) and Sigma-Aldrich (Gillingham, Dorset, UK), respectively. Isoleucine-zipper TRAIL (iz-TRAIL) was expressed and purified in-house^14^. iz-TRAIL is a modified form of rhTRAIL that comprises an isoleucine zipper motif fused to its N-terminus, which enhances its ability to trimerise — a process required for its apoptotic activity, consequently increasing its potency. siRNA sequences targeting RALA (_8 SI03101133), RALB (_1 S100045199; _4 SI00045178; _6 SI03054793; _7 SI03117492), RALGDS (_5 SI03231424), Caspase-8 (_11 SI02661946) and Caspase-9 (_5 SI00299600) were purchased from Qiagen (Crawley, UK). siRNA sequences targeting DR4 and DR5 were purchased from Dharmacon. TRAIL neutralising antibody, Chloroquine and Bafilomycin A1 were purchased from R&D systems (Abingdon, UK), Invivogen (San Diego, CA) and Merck Millipore (Darmstadt, Germany), respectively.

### Cell culture

Authentication and culture of the HCT116, SW620, GP5d, LoVo, LIM1215 and COLO320 CRC cells have previously been described^15,16^. KM12 cells were obtained from the National Cancer Institute-Frederick, Cancer DCT Tumour repository (authentication: SNP arrays, oligonucleotide-base HLA typing, karyotyping and STR) and maintained in Roswell Park Memorial Institute 1640 (RPMI). DiFi, SW403, NCI-498 and HDC8 CRC cells were obtained from Dr. Montagut (Barcelona, Spain) and Prof. Bardelli, respectively^17,18^. HCT116 Caspase-8 wild-type (WT) and null paired cells, DR5 parental and knockout cells, and p53 parental and matched isogenic p53 null CRC cells were a gift from Prof. G. Lahav (Harvard Medical School, Boston)^19^, Prof. M. Rehm (University of Stuttgart) and Prof. B. Vogelstein (Johns Hopkins University, Baltimore), respectively, and were grown in McCoy’s 5 A Modified Medium. All cells were passaged for a maximum of two months. Cell lines were tested for mycoplasma contamination every month using the MycoAlert™ Mycoplasma Detection Kit (Lonza).

### Western blotting

Western blotting and antibodies have previously been described^15^. Antibodies were used in conjunction with a HRP-conjugated anti-rabbit or anti-mouse secondary antibody. β-actin was used as a sample loading control. Details of the antibodies used have been included in Table S1. Western blot images were developed using the G:BOX Chemi XX6 gel doc system (Syngene). Densitometry on western blot images was performed using ImageJ software.

### DR5-DISC IP assay

The DR5-DISC IP assay was performed as previously published^20^. The fully human agonistic DR5 antibody AMG 655 (Conatumumab; Amgen, Thousand Oaks, CA, USA) was conjugated to Dynabeads using the Dynabeads® antibody coupling kit (Life Technologies, Paisley, UK) as per the manufacturer’s instructions. To the cells, 30 μL of AMG 655-conjugated Dynabeads® was added for the indicated time. The cells were lysed in lysis buffer (0.2% NP-40, 20 mM Tris, 150 mM NaCl, 10% glycerol; pH 7.4) supplemented with protease inhibitors. The AMG 655-conjugated Dynabeads® were captured magnetically, washed in lysis buffer, resuspended in Laemmli buffer and analysed by western blotting. Unbound fractions (inputs) were also collected and analysed by western blotting.

### FLAG-co-immunoprecipitation

Protein lysates were prepared using SDS-free RIPA (50 mM Tris, 150 mM NaCl, 1% Triton X-100 and 5 mM EDTA, pH 7.4). Lysates were incubated with anti-FLAG® M2 Magnetic beads (Sigma) overnight and isolated using a magnetic rack. After several washes, beads were resuspended in Laemmli buffer and heated at 95 °C for 5 minutes prior to immunoblot analysis.

### RALB activity assay

GTP-bound RALB was isolated from whole cell lysates using a RALB activation Assay Kit (Merck Millipore). The assay was performed according to the manufacturer’s instructions.

### Expression constructs and transfection

FLAG-tagged WT and constitutively active (G23V) RALB constructs were provided by Prof. Dan Theodorescu (Denver University, Colorado)^21^. Site-directed mutagenesis to generate the other RALB mutants (S28N, C203S) was performed using the KOD Xtreme™ Hot Start DNA Polymerase kit (Merck Millipore). DNA transfection was carried out using XtremeGene-HP reagent (Roche).

### Proteome Profiler Human Apoptosis Array Kit

Array was analysed according to the manufacturer’s instructions (R&D systems, Abingdon). Array densitometry was performed on the duplicate ‘spots’ using ImageJ software. Background intensity was subtracted from raw pixel density values. Normalised pixel density values were represented as a ‘fold-change’ relative to the control values, with error bars representing standard deviation from the mean.

### Flow cytometry

Apoptosis was evaluated using propidium iodide (PI) staining to determine the percentage of cells with DNA content <2 N (sub-G1)^15^. The BD FACS Calibur Flow Cytometer (BD Biosciences) and Cell Quest Pro Software was used to perform PI staining flow cytometry. DR5 cell -surface expression flow cytometry was performed using a phycoerythrin (PE)-conjugated DR5-specific antibody (12-9908-42, ThermoFisher Scientific) and a matched isotype control antibody (12-4714-41, ThermoFisher Scientific). The BD FACS Calibur Flow Cytometer and Cell Quest Pro Software were used to analyse geometric means for cell surface expression. Ten thousand events were counted per experiment for all the flow cytometry experiments.

### High content screening

Annexin V (AV)/PI staining was evaluated using high content screening and performed in a 96-well plate (glass-bottomed plates, Cell Vis) format. Cells were seeded and treated as required (each treatment was performed in triplicate). Post-treatment, 1x AV Binding Buffer (10x concentrate, BD Pharmingen, San Jose, CA, USA), 1:1000 FITC Annexin V (BD Pharmingen), 0.333 µg/ml PI and 1.33 µg/ml Hoechst 33342 (Thermo Fisher Scientific) were added to each well, and the plate was incubated for 20 min at room temperature. High content screening was performed using the ArrayScan™ XTI HCA Reader and the HCS Studio Cell Analysis Software V6.6.0 (ThermoFisher Scientific, Surrey, UK). The CrEST™ X-Light™ Confocal Scan Head (ThermoFisher Scientific) was integrated into the ArrayScan™ Reader, which enabled the capture of fluorescent microscopy images of cells. The ArrayScan™ collected multiple images until a maximum of 2000 cells was imaged per well.

Images were analysed using the HCS Studio Cell Analysis Software V6.6.0 (Thermo Fisher Scientific). Briefly, the software calculated total death by quantifying cells that were AV stained, PI stained or both (cells were identified by nuclear Hoechst staining). This data was then graphed using GraphPad Prism 8.0.

### Caspase-3/7 activity assays

Caspase-3/7-Glo® reagent (25 μl) (Promega, Southampton, UK) was incubated with 5 μg of protein lysate diluted in phosphate buffered saline in a total volume of 50 μl for 45 minutes at room temperature. Luciferase activity was measured using a luminescent plate reader (Biotek Synergy 4 plate reader).

### Crystal violet assays

Cell viability was determined using crystal violet assays, as previously published^22^.

### Immunofluorescence

Cells were seeded into 8-well chambers (ThermoFisher Scientific), incubated overnight and treated as required (each treatment was performed across duplicate chambers within an experiment). Cells were washed in phosphate buffered saline and fixed in 4% paraformaldehyde. Cells were incubated with Alexa-488 labelled Wheat Germ Agglutinin (WGA) (Thermo Scientific Fisher, Cramlington, 1:2000) at room temperature (RT) for 5 minutes. Slides were then washed, permeabilised for 30 minutes (Tris-buffered saline, 10% goat serum (Abcam) and 0.1% Saponin (Sigma)) and left to block overnight (Tris-buffered saline, 5% Goat serum and 0.01% Saponin). Cells were incubated with DR5 (1:100) antibody for 1 hour at RT, followed by washing and were then incubated with Alexa Fluor-568 anti-rabbit secondary antibody (ThermoFisher Scientific, 1:500) for 30 minutes at RT, followed by washing. LAMP-1 (Abcam, 1:50) antibody was used in conjunction with Alexa Fluor-488 anti-mouse secondary antibody (ThermoFisher Scientific, 1:500). Counterstaining and mounting were performed using VECTASHIELD® anti-fade mounting medium with DAPI (Vector Laboratories). Images were captured using a Leica SP8 confocal microscope and a 63x lens at a 2x zoom, 1024 × 1024 frame and a 400 Hz scanning speed. Within an experiment, images were taken using fixed laser settings and exposure times. Images were acquired and processed using Leica Application Suite X (Las X). DR5 colocalisation with TGN46, Calnexin and LAMP1 across separate experiments was investigated using Manders’ colocalisation coefficient (MCC), which is a measure of the co-occurrence of staining intensities in pixels above threshold and was calculated using the JACOP plugin in ImageJ. This enabled the fraction of total DR5 fluorescence colocalising with the fluorescence of the second marker (for e.g. TGN46) to be calculated. A threshold was applied manually to all images using the built-in tool in JACOP. A 90° rotation negative control, where the MCC was re-calculated when the image of the second marker was rotated 90° clockwise, was employed to confirm that co-occurrence of staining was not random^23^.

### Real-time reverse transcription-PCR analysis

RT-PCR analysis was performed using the LightCycler® 480 probes master mix (LightCycler® 480II, Roche). *ACTB* and *GAPDH* were used as housekeeping genes. RNA was isolated using the GeneJET RNA purification kit (ThermoFisher Scientific); quantification and quality control (ratios for 260/280 nm >1.8 and for 260/230 nm >2) were performed using the NanoDrop™ One/One^C^ Microvolume UV-Vis Spectrophotometer (ThermoFisher Scientific). cDNA synthesis was performed using the Moloney murine leukaemia virus-based reverse transcriptase kit (Invitrogen, ThermoFisher Scientific). Catalogue numbers for real-time ready probes (Roche) are as follows: *RALA*- 115411, *RALB*- 148403, *TNFRSF10B*- 101236, *ACTB*- 101125, *GAPDH*- 101128 and *RALGDS*- 145711. PCR primers for *FLIP*_*L*_ and *FLIP*_*S*_ were purchased from Eurofins Genomics and were used in conjunction with LightCycler® 480 SYBR Green I Master mix (Roche). (*FLIP*_*L*_: Forward: 5′-CCT AGG AAT CTG CCT GAT AAT CGA, Reverse: 5′-TGG GAT ATA CCA TGC ATA CTG AGA TG; *FLIP*_*S***:**_ Forward: 5′-GCA GCA ATC CAA AAG AGT CTC A, Reverse: 5′-ATT TCC AAG AAT TTT CAG ATC AGG A).

### siRNA transfections

siRNA transfections were carried out using HiPerfect (Qiagen) as previously described^15^.

### Analysis of clinical data

The stage II/III CRC patient cohorts GSE103479 (*n* = 156)^24^, the GSE39582 (*n* = 481)^25^ and GSE14333 (*n* = 185)^26^ datasets, and their consensus molecular subtypes (CMS) and Colorectal Cancer Intrinsic Subtypes (CRIS) classification have been described^24^. GSE59857 is a dataset comprising transcriptomic data for 155 established CRC cell lines. The COREAD dataset comprises protein abundance data for 50 CRC cell lines^27^. For survival analyses, the median value for *RALB* expression values was identified using the probe set corresponding to *RALB* (ADXECAD.28315_at and 202100_at). Patient samples were then allocated to one of two categories: *RALB* low and *RALB* high. Survival curves, comparing *RALB* low (grey) with *RALB* high (black) expression groups, were estimated with the Kaplan Meier method and compared by the log-rank test, using GraphPad Prism version 8 for Windows, GraphPad Software, La Jolla, CA, USA, www.graphpad.com. To assess RALB protein expression in clinical samples, we accessed fresh frozen matched CRC primary and normal tissues, collected during 2001–2002 at the Belfast City Hospital (REC49/01). This work was approved by the School of Medicine, Dentistry and Biomedical Science ethics committee (19.12V1). Consent was obtained from all patients.

### Statistical analysis

Statistical significance was calculated from distinct technical replicates, by Student’s *t*-test (two-tailed, two-sample equal variance on unpaired data), one-way or two-way ANOVA in GraphPad Prism 8, unless specified otherwise. One-way ANOVA tests were used where multiple groups were compared to each other, whereas two-way ANOVA tests were performed to compare two factors (for e.g. siRNA and drug) across multiple groups. Multiple comparison analyses (for 1- and 2-way ANOVA tests) were performed using GraphPad Prism 8. Graphs were plotted as means with error bars representing standard deviation from the mean. Statistical significance is denoted as follows: **** = *p* < 0.0001, *** = *p* < 0.001, ** = *p* < 0.01, * = *p* < 0.05, ns = *p* > 0.05. Experimental phenotypes were confirmed in at least three independent experiments, unless specified otherwise.

## Results

### RALB is required for the survival of *KRAS*MT CRC cells

**Fig. 1:**
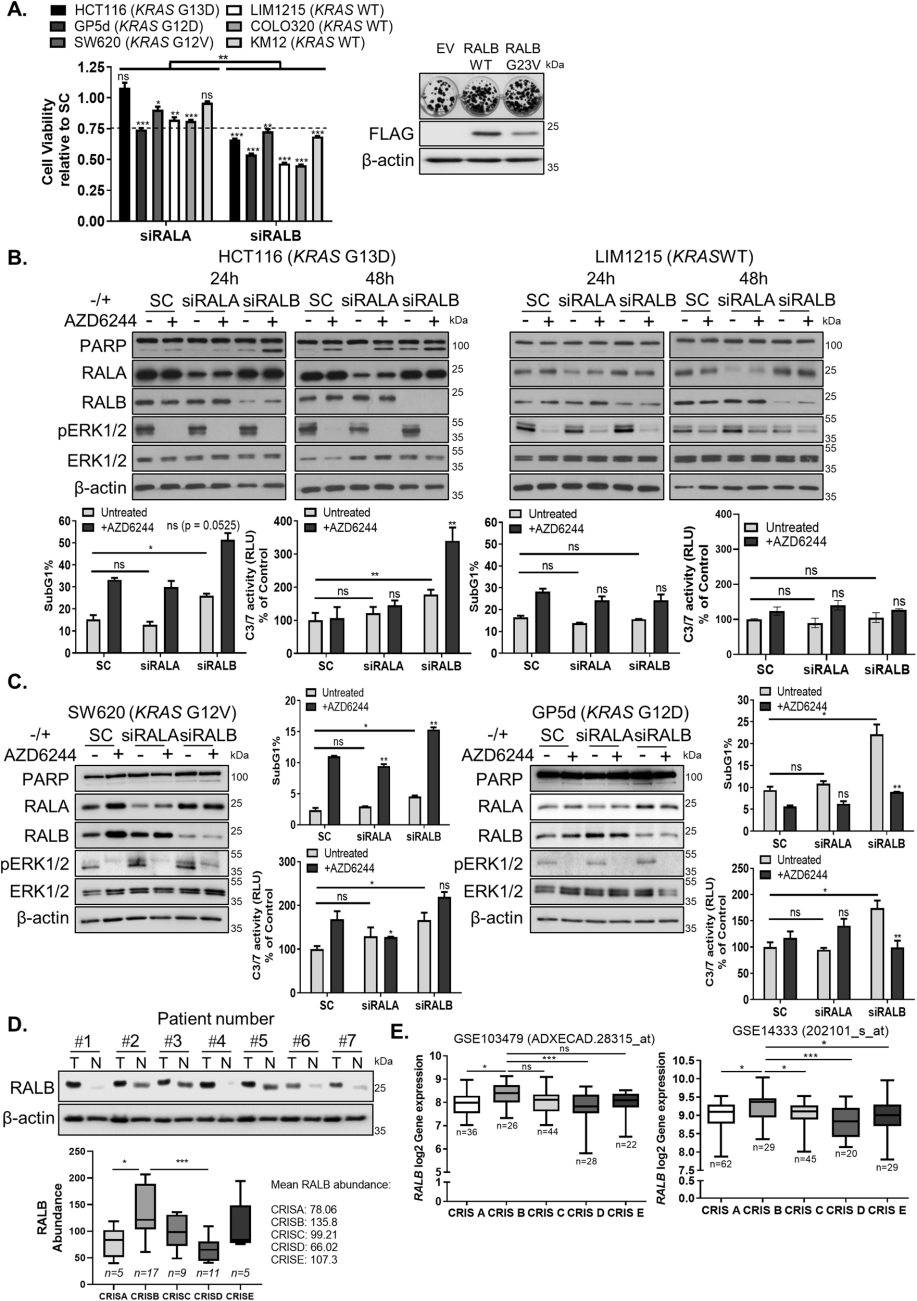
*RAS*MT CRC cells are dependent on RALB for survival. **A** Left: Crystal violet cell viability assay in *KRAS*MT and *KRAS*WT cells following transfection with 10 nM scrambled control (SC), RALA (siRALA) or RALB (siRALB) siRNA for 72 h. Cell viability values are presented relative to SC and the dashed line represents a 25% decrease in viability. Right: Clonogenic survival assay in HCT116 cells following transfection with 1 μg empty vector (EV), RALB wild-type (WT) or RALBG23V constructs and incubation for 14 days. Western blot (WB) analysis of FLAG-tag carried out in HCT116 cells transfected with EV, RALB-WT and RALBG23V constructs. β-actin was used as a loading control. **B** Top: WB analysis of PARP, RALA, RALB, pERK1/2^T202/Y204^ and ERK1/2 expression in HCT116 and LIM1215 cells, following transfection with SC, siRALA or siRALB and co-treatment with 1 μM AZD6244 for the indicated time. Bottom: Apoptosis was assessed by propidium iodide (PI) flow cytometry and Caspase-3/7 activity levels in HCT116 and LIM1215 cells, following transfection with SC, siRALA or siRALB and co-treatment with 1 μM AZD6244 for 24 h. **C** SW620 and GP5d cells were transfected with SC, siRALA or siRALB and co-treated with 1 μM AZD6244. PARP, RALA, RALB, pERK1/2^T202/Y204^ and ERK1/2 expression was determined by WB and β-actin was used to assess equal loading. Apoptosis was assessed by PI flow cytometry and by measuring Caspase-3/7 activity. **D** Top: WB analysis showing RALB expression in matched CRC (T) and normal (N) tissues. Equal loading was assessed by analysing β-actin expression. Bottom: Boxplots representing RALB protein abundance across CRIS subgroups in the COREAD dataset. **E** Boxplots representing the log2 gene expression values for *RALB* (probes ADXECAD.28315_at and 202101_s_at) across CRIS subgroups in GSE103479 and GSE14333 clinical datasets. Numbers underneath the boxplots (*n*) indicate the sample number per group.

Previous data from our lab have shown that RALA, but not RALB, regulates migration of *KRAS*MT CRC cells^28^. We determined whether these two structurally related GTPases^29^ have similar roles in regulating the survival of *KRAS*MT CRC cells (Fig. 1A). Although silencing of RALA resulted in minor decreases in the viability of *KRAS*MT and WT CRC cells, siRALB was found to affect the viability of these cells to a greater extent. This was confirmed using additional siRNA sequences against *RALB* (Fig. S1A). Furthermore, overexpression of WT-RALB or constitutively active G23V-RALB markedly increased the colony-forming ability of *KRAS*MT CRC cells (Fig. 1A). Importantly, siRALB resulted in cell death only in *KRAS*MT but *not* in WT cells as determined by PARP cleavage, sub-G_1_ levels and Caspase-3/7 activation (Fig. 1B, C and Fig. S1A–C). siRALA did not induce cell death in *KRAS*MT CRC cells (Fig. 1B, C and Fig. S1D).

*KRAS*MT cancers are unresponsive to MEK inhibition (MEKi)^30,31^. A previous study has shown a role for RALB in mediating resistance to knockdown of *NRAS*V12 in AML^32^. We therefore hypothesised that RALB has a potential role in regulating resistance to MEKi in *KRAS*MT CRC. Co-treatment of RALB siRNA with the MEKi AZD6244 resulted in significant increases in cell death, determined by PARP cleavage, increased sub-G_1_ levels and Caspase-3/7 activity in HCT116 cells (Fig. 1B and Fig. S1B). This was not observed in *KRAS*WT CRC cells (Fig. 1B and Fig. S1C). We extended these studies to include a panel of *KRAS*MT (SW620, GP5D and LoVo) cells. In contrast to the HCT116 cells, addition of AZD6244 to siRALB resulted in no significant increases in cell death compared to the effect of siRALB alone in these cells (Fig. 1C and Fig. S1D). Furthermore, co-treatment of AZD6244 with siRALA did not increase cell death in *KRAS*MT or WT cells. No consistent changes in expression levels of pERK1/2 were observed following siRALB or siRALA. Collectively, these results indicate that RALB, but *not* RALA, regulates survival of *KRAS*MT but *not KRAS*WT CRC cells.

### Clinical relevance of RALB in CRC

Analysis of RALB expression revealed higher RALB levels in tumour compared to the matched normal patient tissues (Fig. 1D). To improve CRC patient management in the context of precision medicine, a recent study has identified five CRIS subtypes, with *KRAS* frequently mutated in CRIS-A, CRIS-B and CRIS-E^13^. Interestingly, in three publicly available CRC transcriptomic patient datasets (GSE39582/GSE103479/GSE14333)^24–26^, we found significantly higher levels of *RALB* gene expression in the poor prognostic CRIS-B subtype, compared to the levels observed in any of the other CRIS subgroups (Fig. 1E and Fig. S2A). Similar findings were made in the publicly available 155 CRC cell lines transcriptomic dataset GSE59857 (Fig. S2B) and the 50 CRC cell lines protein abundance COREAD dataset (Fig. 1D, lower)^27^. To assess the prognostic value of *RALB*, we interrogated an early-stage II/III CRC dataset (GSE103479). Analysis of *RALB* mRNA expression (LOW versus HIGH-*RALB*) in this dataset showed that, although patients with high *RALB* expression had a decreased overall survival compared to patients with low *RALB* expression (HR = 1.554; 95%-CI: 0.7701–3.135), this did not reach statistical significance (*p* = 0.21) (Fig. S2C). Similar data were obtained using a second early-stage dataset (HR = 1.339; 95%-CI: 0.7889–2.489; *p* = 0.25) (Fig. S2C). Collectively, these data indicate that *RALB* is highly expressed in CRIS-B CRC, a poor prognostic subgroup in need of novel treatment strategies.

### RALB depletion induces apoptosis in a Caspase-8-dependent manner

**Fig. 2:**
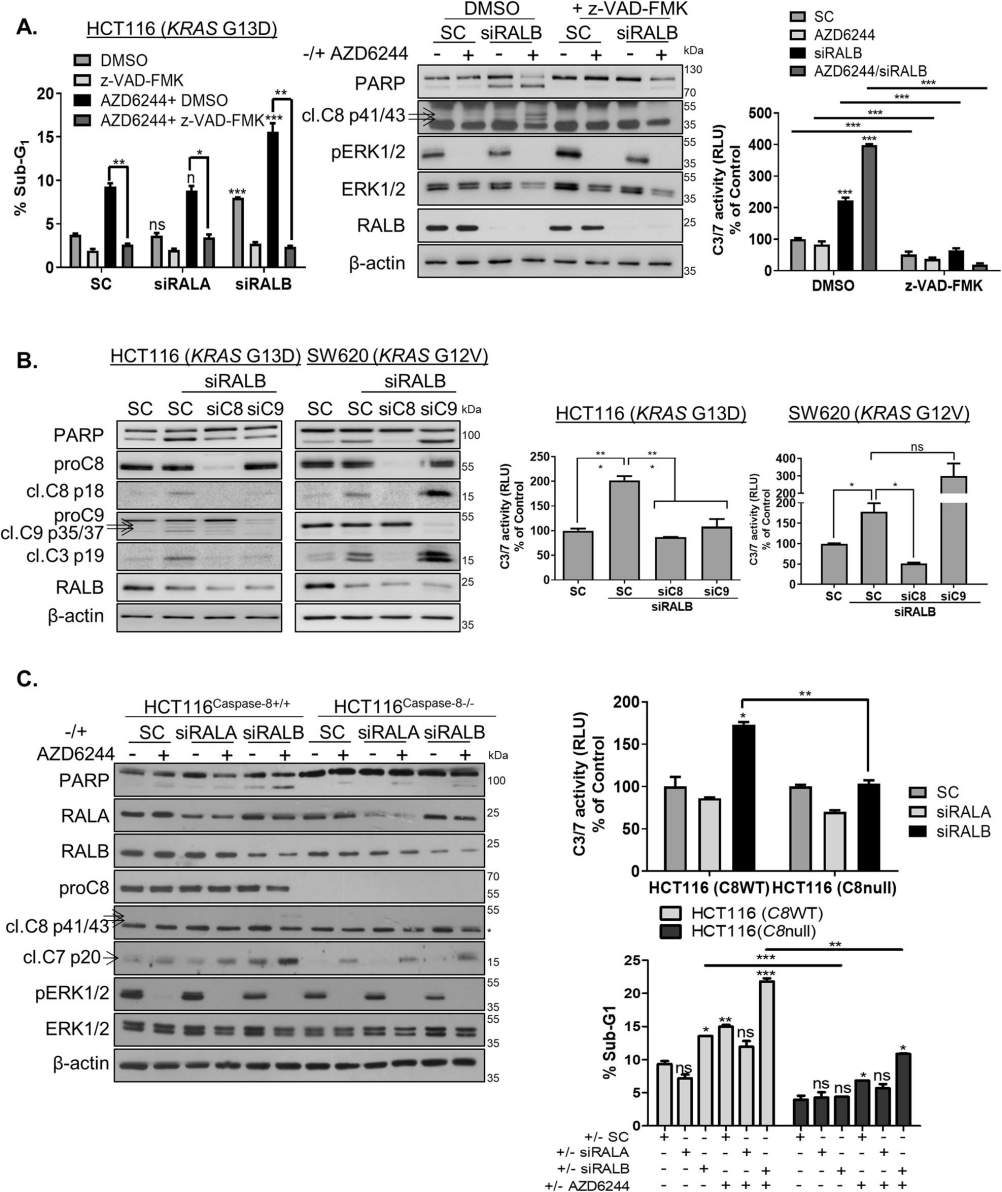
RALB silencing induces Caspase-8-dependent apoptosis. **A** HCT116 cells were transfected with 10 nM SC, siRALA or siRALB and pre-incubated with DMSO or 20 μM of the pan-caspase inhibitor, z-VAD-FMK for 3 h followed by treatment with 1 μM AZD6244 for 24 h, following which apoptosis was assessed by PI flow cytometry (left), WB analyses for PARP and cleaved Caspase-8 (middle) and Caspase-3/7 activity assays (right). **B** CRC cells were transfected with 10 nM C8 or C9 siRNA for 24 h and were thereafter transfected with 10 nM RALB siRNA for 24 h. Apoptosis was assessed by WB analysis for cleaved Caspase-8, Caspase-9 and Caspase-3 (left) and Caspase-3/7 activity (right). **C** Paired CRISPR HCT116^Caspase-8+/+^ and HCT116^Caspase-8-/-^ cells were transfected with 10 nM SC, RALA or RALB and co-treated with 1 µM AZD6244 for 24 h. Apoptosis was determined by WB for PARP, Caspase-8 (* indicates a non-specific band) and Caspase-7 (left), Caspase-3/7 activity levels (right, top) and PI flow cytometry (right, bottom).

A previous study in *KRAS*MT NSCLC found that RALB inhibits anchorage-independent growth in a p53-dependent manner^33^. In contrast to this study, no marked differences in cell death were found following siRALB in our p53-WT parental and isogenic p53 null HCT116 cells (Fig. S3A). Caspase-dependent apoptosis following siRALB was assessed using the pan-caspase inhibitor z-VAD-FMK, which completely attenuated increased sub-G_1_ levels, activation of PARP and Caspases-3/7 in *KRAS*MT cells (Fig. 2A and Fig. S3B). To investigate further the relative importance of the extrinsic and intrinsic apoptotic pathways in mediating siRALB-induced apoptosis, CRC cells were co-transfected with siRALB and siCaspase-8 or siCaspase-9. Notably, co-silencing of Caspase-8 with RALB resulted in inhibition of siRALB-induced PARP and Caspase-3/7 activity in both *KRAS*MT HCT116 and SW620 cells (Fig. 2B). These results were confirmed using the Caspase-8 null HCT116 cells (Fig. 2C). We previously reported that HCT116 cells behave in a type II manner, with the extrinsic apoptotic pathway requiring mitochondrial amplification via the intrinsic apoptotic pathway in order to commit to apoptosis^34^. This model can explain why siCaspase-9 also decreased siRALB-induced apoptosis in the HCT116 cells (Fig. 2B). Collectively, these data suggest that the apoptosis induced by siRALB proceeds via a Caspase-8-mediated activation of the extrinsic apoptotic pathway.

### RALB depletion increases total and cell-surface DR5 levels

**Fig. 3:**
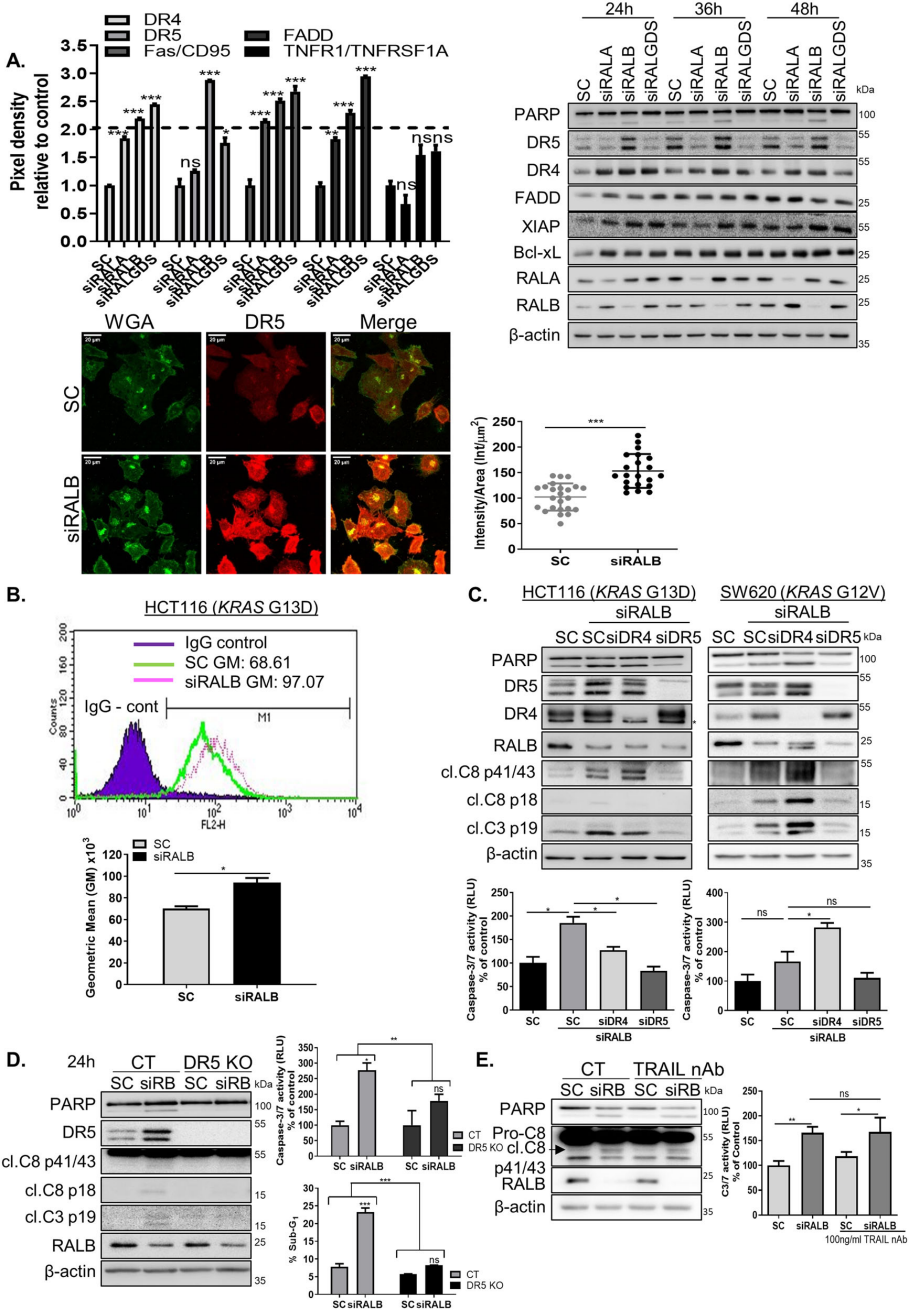
RALB silencing regulates DR5 expression levels. **A** Top Left: HCT116 cells were transfected with 10 nM SC, siRALA, siRALB or siRALGDS for 24 h, following which cells were harvested for protein extraction. Protein was subsequently analysed using a human apoptosis array. Densitometry was performed on the array panels using ImageJ software, and results for DR4, DR5, Fas/CD95, FADD and TNFR1/TNFRSF1A are shown. Top right: PARP, DR5, DR4, FADD, XIAP, Bcl-xL, RALA and RALB levels in CRC cells following RALA, RALB and RALGDS silencing for the indicated times. Bottom left: Wheat germ agglutinin (WGA), a lectin that binds *N*-acetylglucosamine post-translational modifications on membrane receptors, was used as a plasma membrane marker. Fixed cells were stained with Alexa488-WGA prior to permeabilisation to minimise the staining of glycosylated proteins in the Golgi apparatus. A single confocal image was collected of the Alexa488-WGA stained membrane (green) alongside immunostained endogenous DR5 (red). Laser settings were kept constant between the samples to enable the comparison of membrane-associated DR5 staining in control (SC) and siRALB-treated cells. Bottom right: >20 cells were scored for intensity in ImageJ. The intensity was normalised to cellular area and plotted. The data are representative of three independent experiments. **B** CRC cells were transfected with 10 nM SC or siRALB for 24 h and DR5 cell-membrane expression was assessed by flow cytometry using a DR5-specific phycoerythrin-conjugated mAb. Expression was compared with an isotype-matched control antibody (IgG control). Geometric means (GM) for fluorescence intensity were plotted and significance was analysed using an unpaired *t*-test. **C** CRC cells were transfected with 10 nM siRNA targeting DR4 or DR5 for 24 h and were thereafter transfected with 10 nM RALB siRNA for 24 h. Apoptosis was assessed using WB for PARP, cleaved Caspase-8 and Caspase-3 (top) and Caspase-3/7 activity (bottom). (Asterisk next to the western blot indicates an unspecific band). **D** Paired CRISPR parental (control = CT) and DR5 knockout (KO) cells were transfected with 10 nM SC or RALB siRNA (siRB) for the indicated time. Apoptosis was determined by WB for PARP, Caspase-8 and Caspase-3 (left), Caspase-3/7 activity (right, top) and PI flow cytometry (right, bottom). **E** WB analysis for PARP and Caspase-8 (left) and Caspase-3/7 activity (right) of the effect of TRAIL neutralising antibody (nAb) treatment (100 ng/ml) on cell death in HCT116 cells transfected for 48 h with 10 nM SC or siRALB.

**Fig. 4:**
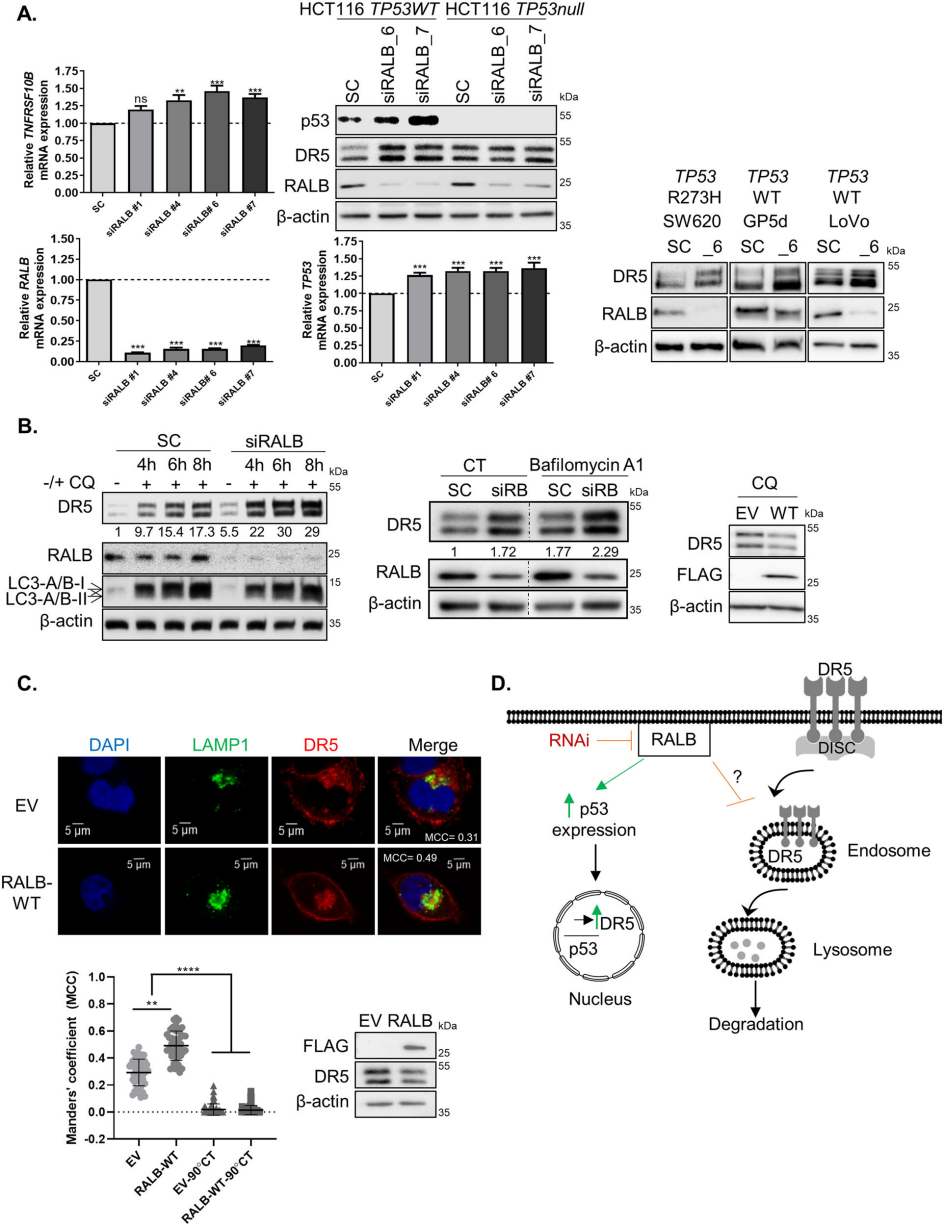
DR5 expression is regulated by RALB through lysosomal degradation and p53-mediated transcription. **A** Left: HCT116 cells were transfected with 10 nM of SC or 4 different siRNA sequences targeting RALB for 24 h. *TNFRSF10B* (upper) and *RALB* (lower) mRNA was quantified using RT-PCR. Raw values were normalised to the expression of housekeeping genes *ACTB* and *GAPDH* and were analysed using the ΔΔCT method. mRNA levels presented are relative to SC. Middle top: HCT116 p53 wild-type (WT) and null cells were transfected with either 10 nM SC or siRALB (two different sequences) for 24 h and the protein expression of p53, DR5 and RALB was determined using WB. Middle bottom: HCT116 cells were transfected with 10 nM of SC or four different siRNA sequences against RALB for 24 h. *TP53* mRNA was quantified using RT-PCR. Analysis was performed as described above. Right: SW620, GP5d and LoVo cells were transfected with 10 nM SC or RALB siRNA (_6) for 24 h and DR5 expression was determined by WB. **B** Left: HCT116 cells were treated with siRNA targeting either SC or RALB for a total transfection time of 24 h. Cells were treated with 20 µM Chloroquine (CQ) for 4, 6 or 8 h. The expression of DR5, RALB, LC3-A/B and β-actin was analysed using WB. The numbers at the bottom of the WB panel represent densitometry performed on the DR5 blot using ImageJ. Middle: HCT116 cells were transfected with siRNA targeting either SC or siRALB and were treated with 50 nM Bafilomycin A1 for 3 h for a total transfection time of 24 h. The expression of DR5, RALB and β-actin was analysed using WB. The numbers at the bottom of the WB panel represent densitometry performed on the DR5 blot using ImageJ. Right: HCT116 cells were transfected with 1 µg of a WT FLAG-tagged RALB construct for 24 h followed by treatment with 20 µM Chloroquine for a further 24 h and DR5 and FLAG-tag expression was determined by WB. **C** Top: HCT116 cells were transfected with either empty vector (EV) or FLAG-RALB WT for 24 h. Cells were then fixed, permeabilised, blocked and stained with a lysosomal marker LAMP1, and DR5 antibody. Bottom left**:** Manders’ colocalisation coefficient (MCC) was calculated using ImageJ to measure the fraction of total DR5 fluorescence overlapping with LAMP1 fluorescence for each treatment and is presented in the ‘Merge’ image rounded to two decimal places. Image presented is representative of three independent experiments. Additional cells are presented in Fig. S5J. The MCC was calculated for over 50 cells across each treatment group over three independent experimental repeats and the analyses are presented in the graph. A 90° rotation negative control was employed to ensure colocalisation was not random. A Kruskal Wallis one-way ANOVA with a multiple comparisons test was performed in Prism to statistically analyse the data. Error bars represent standard deviation from the mean. Bottom right: HCT116 cells were transfected with either EV or FLAG-RALB WT for 24 h, and FLAG and DR5 expression was determined by WB. **D** Schematic of the role of RALB in regulating DR5 expression levels.

Given the Caspase-8-dependent manner of siRALB-induced apoptosis, the expression of FLIP_L/S_, an endogenous inhibitor of Caspase-8 activation, was assessed (Fig. S3C). Although *FLIP*_*L*_ mRNA levels were found to be slightly increased in the siRALB-transfected cells, FLIP_L_ protein levels were unaffected. To further investigate the upstream mechanisms specifically involved in siRALB-induced apoptosis, a protein array was used to assess simultaneously the expression of 35 human apoptosis-related proteins in *KRAS*MT cells, following transfection with siRALB, siRALA or siRALGDS (Fig. 3A and Fig. S4A–F). Notably, siRALB induced both pro-apoptotic and anti-apoptotic signalling responses. Significantly (2-fold cut-off; *p* < 0.05) increased levels of DR5/TRAIL-R2, XIAP and the anti-apoptotic Bcl2-family member Bcl-xL were found following siRALB; these did not change in the siRALA-transfected cells. We validated these array results by western blotting (Fig. 3A). Importantly, only DR5 levels increased dramatically as early as 24 h following transfection with siRALB but not in the siRALA or siRALGDS-transfected cells. DR4 was upregulated in both RALA and RALB knockdown cells. DR5 upegulation was confirmed with three additional siRNA sequences (Fig. S5A) and in three additional *KRAS*MT CRC cell lines (Fig. 4A). The DR5 upregulation observed in RALB-silenced cells was not an indirect result of caspase activation, as inhibition of apoptosis using the Caspase-8 null HCT116 cells failed to prevent DR5 upregulation in RALB-silenced cells (Fig. S5B). Interestingly, silencing of RALB did not increase DR5 levels in *KRAS*WT cells (Fig. S5C). Using immunofluorescence confocal microscopy, we further showed that siRALB resulted in a significant increase in DR5 staining intensity (Fig. 3A and Fig. S5D). Furthermore, DR5 cell-surface expression analysis in siRALB-transfected cells confirmed these results (Fig. 3B and Fig. S5E). Previous studies have shown that DR5 can signal from the intracellular ER-Golgi compartment, independently of TRAIL^35^. We therefore assessed the impact of RALB depletion on colocalisation of the Golgi marker TGN46 and the Endoplasmic Reticulum marker Calnexin with DR5, and found no increases in colocalisation of the two markers with DR5 when RALB was silenced (Fig. S5F, G). Although no significant negative correlations were observed between RALB and DR5 protein levels within the CRIS-B group, we found that the DR5/RALB ratio was the lowest in the CRIS-B group compared to the other CRIS subgroups (Fig. S2D–F).

### siRALB-induced apoptosis is DR5-dependent

To investigate whether DR4/TRAIL-R1 contributes in a similar manner to DR5 in regulating siRALB-induced apoptosis, specific siRNAs were used to decrease the expression of each receptor (Fig. 3C). Depletion of DR4 slightly reduced the Caspase-3 cleavage and Caspase-3/7 activity observed following siRALB in HCT116 cells (Fig. 3C). In contrast, co-silencing of DR4 and RALB in the SW620 cells did not decrease apoptosis compared to the levels observed with siRALB alone. Importantly, siDR5 completely abrogated siRALB-induced PARP, Caspase-3 cleavage and Caspase-3/7 activity in both *KRAS*MT cell lines (Fig. 3C). These data were validated using WT and DR5-knockout HCT116 cell lines (Fig. 3D and Fig. S4G). Canonically, DR5-mediated activation of Caspase-8 and apoptosis is triggered following ligation of its ligand TRAIL, which is normally expressed on the cell surface of various immune cells^36^. Notably, co-incubation with a TRAIL neutralising antibody failed to rescue siRALB-induced apoptosis in *KRAS*MT CRC cells (Fig. 3E). Taken together, these results would suggest a causal role for DR5, but not for DR4, in the cell death following siRALB in *KRAS*MT CRC.

### RALB reduces DR5 expression through enhancing lysosomal degradation

Previous studies have shown that DR5 may be transcriptionally upregulated by p53^37^ and that depletion of RAL-GTPases can stabilise p53^33^. We therefore analysed if the increased DR5 levels observed following siRALB was a result of transcriptional changes. Quantitative-PCR analysis showed that silencing of RALB, and not RALA, resulted in minimal increases in *TNFRSF10B* mRNA levels (1.19–1.46 fold) (Fig. 4A and Fig. S5H). No changes in expression levels of CHOP or the NFκB-inhibitor alpha (IKBα) protein, key transcriptional activators of DR5^38,39^, were observed following siRALB (Fig. S5I). In addition, sip65 did not affect basal DR5 levels (Fig. S5I). Silencing of *RALB* resulted in minimal increased (1.26–1.36-fold) *TP53* mRNA and protein levels (Fig. 4A). Although siRALB-induced DR5 upregulation was partially abrogated in the *TP53* null HCT116 model (Fig. 4A), we also found strong DR5 increases following siRALB in SW620 cells expressing the clinically relevant DNA-binding-defective mutant p53-R273H (Fig. 4A and Fig. S5E). These results indicate that siRALB-induced DR5 upregulation can occur independently of p53.

Beyond a regulator of transcription factors, RALB is involved in vesicular trafficking and activation of the autophagosome/lysosome assembly, and this function may contribute to the accumulation of DR5 in RALB knockdown cells (Fig. 3A)^40^. Furthermore, a number of previous studies have shown autophago-lysosomal regulation of DR5 levels^41^. Therefore, we next determined the impact of RALB depletion on DR5 lysosomal degradation, using the lysosomal inhibitor chloroquine (CQ) (Fig. 4B). In agreement with other studies, we found that CQ resulted in increased DR5 levels^42^. Furthermore, siRALB markedly enhanced the upregulation of DR5 induced by CQ, while there was no further change in the autophagy marker LC3. Similar data were obtained using the lysosomal inhibitor Bafilomycin A1 (Fig. 4B)^43^. In addition, transient overexpression of RALB abrogated the increased DR5 expression levels following CQ treatment (Fig. 4B). Importantly, overexpression of RALB resulted in increased colocalisation of endogenous DR5 with LAMP1, as indicated by the MCC values, demonstrating that RALB regulates lysosomal localisation of DR5 (Fig. 4C and Fig. S5J). The proteasomal inhibitor MG132 did not affect siRALB-induced DR5 levels (Fig. S5K, left panel). We also compared the effect of siRALB on DR5 stability using the protein synthesis inhibitor cycloheximide. The result showed that the degradation rate of DR5 was unchanged in siRALB-transfected cells (Fig. S5K, right panel). Collectively, these data indicate that RALB can regulate DR5 protein levels by altering its trafficking to the lysosomal compartment for degradation (Fig. 4D).

### RALB depletion sensitises *KRAS*MT CRC cells to rhTRAIL

**Fig. 5:**
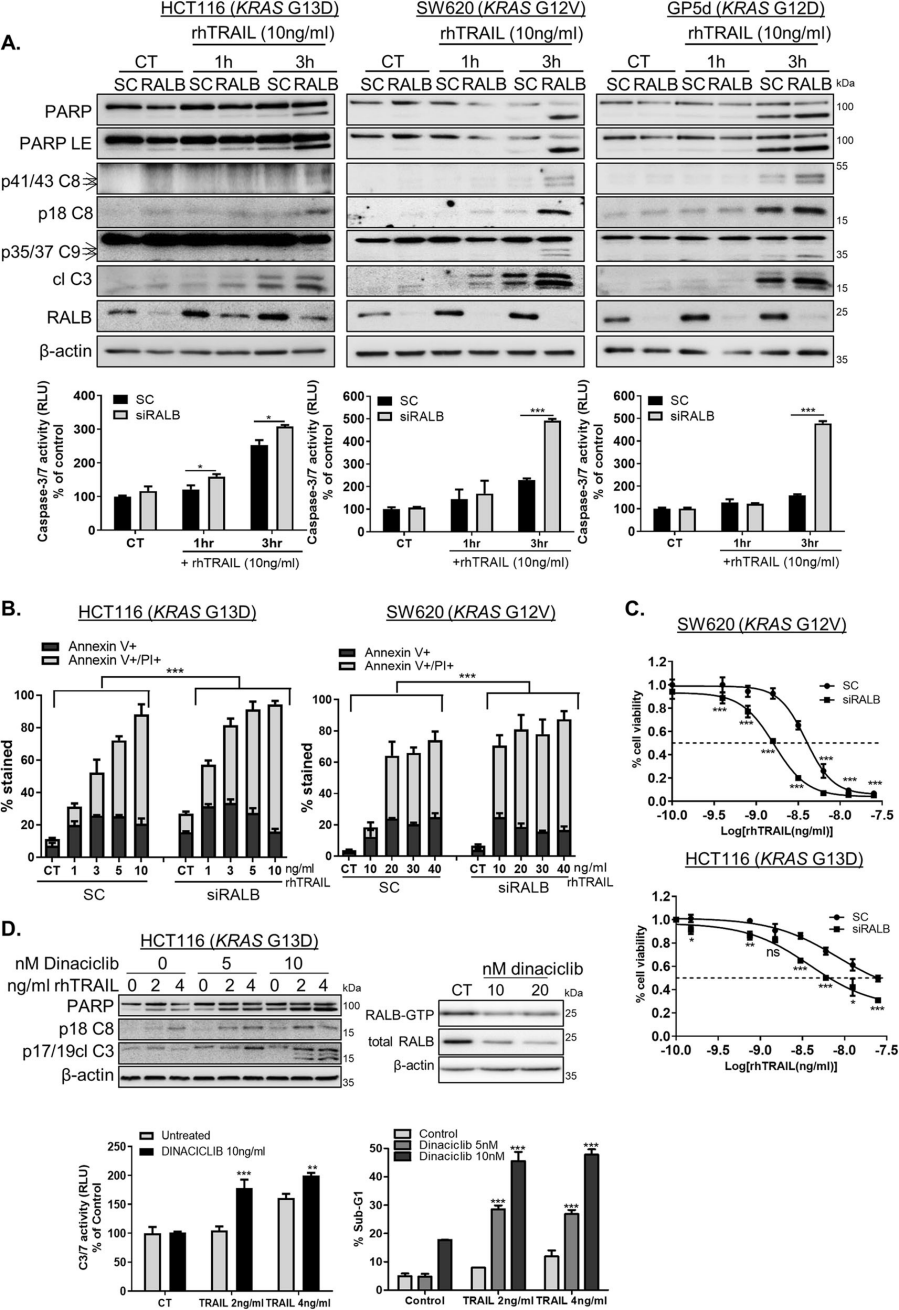
RALB inhibition enhances TRAIL-induced apoptosis in *KRAS*MT CRC. **A** CRC cells were transfected with 10 nM RALB siRNA for 24 h prior to treatment with rhTRAIL for the indicated time, (CT = control, untreated sample). Apoptosis was determined by using WB analysis for PARP, cleaved Caspase-8, -9 and -3 levels (top) and Caspase-3/7 activity (bottom). LE = long exposure. **B** HCT116 and SW620 cells were transfected with either SC or siRALB for 24 h followed by rhTRAIL treatment for a further 24 h. Apoptosis was assessed using Annexin V/propidium iodide (PI) staining by high-content screening. The graph indicates the percentage of positive stained cells. **C** HCT116 and SW620 CRC cells were transfected with siRALB or control siRNAs and then treated with the indicated doses of rhTRAIL for 72 h. An MTT assay was used to evaluate cell viability, which is presented relative to SC control on the graphs. The dashed line indicates a 50% change in cell viability. **D** HCT116 cells were treated with Dinaciclib for 24 h, prior to treatment with rhTRAIL for 24 h. WB analysis for PARP, cleaved Caspase-8 and -3 (Left top), Caspase-3/7 activity assays (left bottom) and PI flow cytometry analysis of the sub-G_1_ apoptotic population (right bottom). HCT116 cells were treated with Dinaciclib for 48 h and expression of active (GTP-bound) and total RALB was evaluated using WB. CT refers to an untreated sample (right top).

A number of studies have indicated that cell-surface TRAIL-receptor expression levels correlate with TRAIL-resistance/sensitivity^44,45^. As our results described above show that RALB depletion increases total and cell-surface DR5 levels, we hypothesised that RALB may be additionally involved in regulating sensitivity to TRAIL treatment in *KRAS*MT CRC. Notably, rhTRAIL treatment markedly increased PARP, Caspase-8, -9 and -3 cleavage in RALB-silenced *KRAS*MT cells (Fig. 5A). These results were confirmed quantitatively using Caspase-3/7 activity assays and Annexin V/propidium iodide staining (Fig. 5A, B). In addition, cell viability of RALB-depleted, rhTRAIL-treated *KRAS*MT cells was significantly decreased compared with control siRNA-transfected cells (Fig. 5C). Similar effects were observed in a panel of *KRAS*MT CRC cells and using two additional *RALB* siRNA sequences (Fig. S6A, B). Notably, RALB expression was acutely increased in 6/7 *KRAS*MT cell lines in response to rhTRAIL treatment (Fig. 5A and Fig. S6A).

The results described above clearly indicate that RALB downregulation sensitises *KRAS*MT CRC cells to rhTRAIL-induced apoptosis. Previous studies have shown that Dinaciclib, a broad spectrum cyclin-dependent kinase inhibitor, is an efficient suppressor of CDK5-mediated activation of RALB^32^. In HCT116 cells treated with Dinaciclib, RALB activity was downregulated (Fig. 5D). In agreement with the results in RALB-depleted cells, Dinaciclib potently increased rhTRAIL-induced apoptosis (Fig. 5D). Similar results were obtained in the *KRAS*MT SW620 and GP5d cells (Fig. S6C). Collectively, these findings indicate that RALB-targeted agents may be highly effective when used in conjunction with DR5 agonists to treat *KRAS*MT CRC.

### RALB associates with the DR5-DISC in a Caspase-8-dependent manner

**Fig. 6:**
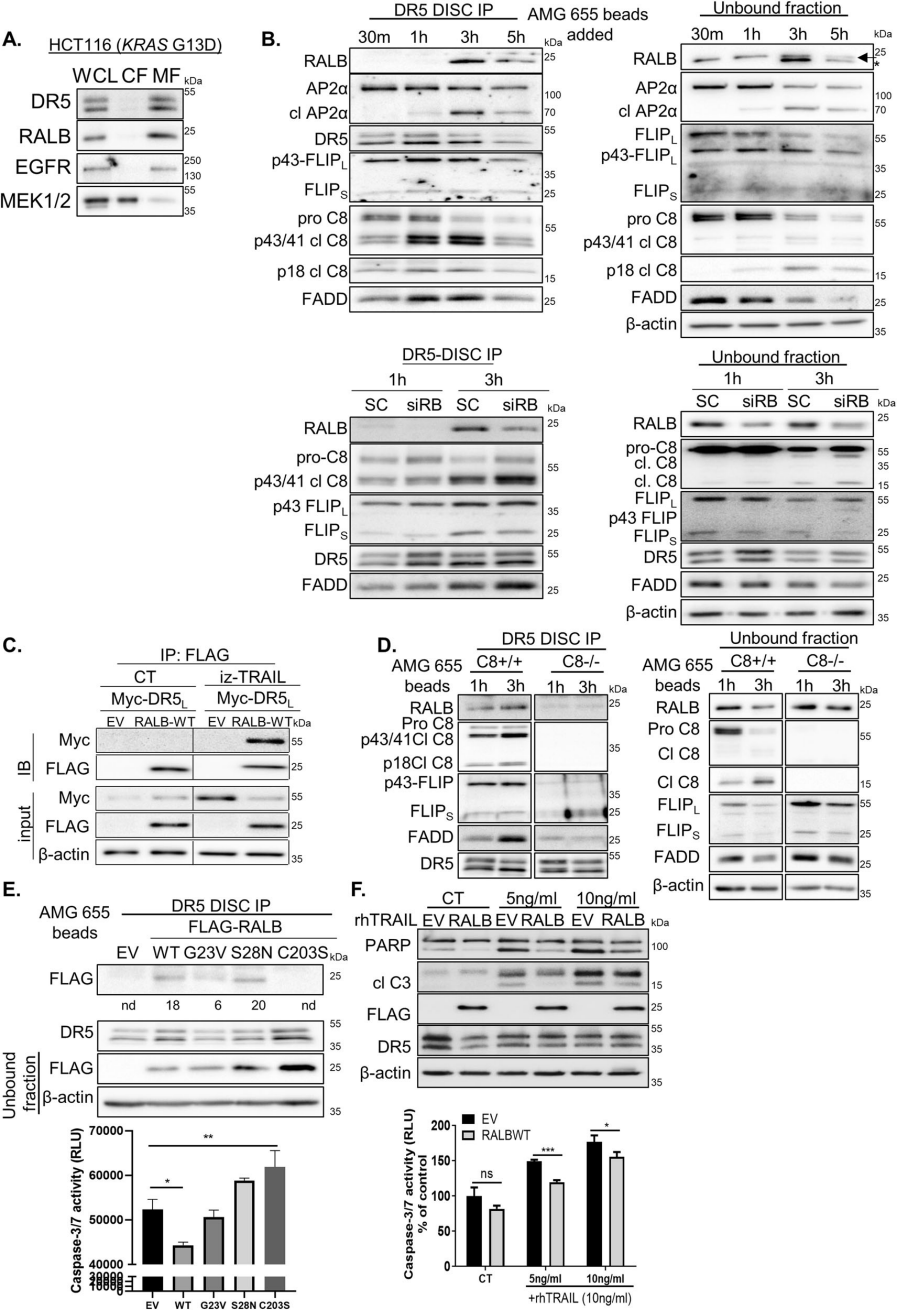
rhTRAIL treatment results in association of RALB with the DR5-DISC (DR5-bound death-inducing signalling complex). **A** Membrane (MF) and cytosolic (CF) fractions were isolated from HCT116 cells, and equal amounts of protein were immunoblotted for DR5, RALB, EGFR and MEK1/2. WCL = whole-cell lysate **B** Top: WB analysis of DR5-DISC IP performed in HCT116 cells. The DR5-DISC was captured magnetically after the addition of AMG 655-conjugated beads to cells for the indicated times. Co-immunoprecipitation of RALB, AP2α, FLIP, FADD and Caspase-8 was assessed and pull-down of DR5 confirmed. Protein expression in the non-DISC recruited fraction (unbound fraction) was also analysed. Bottom: HCT116 cells were transfected with either SC or siRALB for 24 h. A DR5-DISC IP was performed as described above, following a 1 h and 3 h incubation with AMG 655-conjugated beads. Co-immunoprecipitation of RALB, FLIP, FADD and Caspase-8 was assessed and pull-down of DR5 confirmed. Protein expression in the non-DISC recruited fraction (unbound fraction) was also analysed. **C** HCT116 cells were transiently transfected with empty vector (EV), FLAG-RALB WT and C-terminal Myc-tagged long isoform of DR5 (DR5_L_) for 24 h followed by incubation with 0.5 ng/ml isoleucine zipper (iz)-TRAIL for 5 h. FLAG-RALB was immunoprecipitated using Anti-FLAG® M2 dynabeads and the expression of Myc-tag and FLAG-tag was determined by WB. Protein expression for Myc and FLAG were also determined by WB in the input lysates. **D** HCT116^Caspase-8+/+^ and HCT116^Caspase-8−/−^ cells were incubated with AMG 655-conjugated dynabeads for 1 h and 3 h. The DR5-DISC was isolated and expression of RALB, Caspase-8, FLIP, FADD and DR5 was determined by WB. Protein expression in the unbound fraction was also analysed. **E** Top: HCT116 cells were transiently transfected for 24 h with 1 µg of EV, FLAG-RALB WT, FLAG-RALB G23V (constitutively active), FLAG-RALB S28N (dominant negative) or FLAG-RALB C203S (geranyl-geranylation deficient) for 24 h. The DR5-DISC was captured 3 h after the addition of AMG 655-conjugated beads to cells. Co-immunoprecipitation of FLAG-RALB was assessed and pull-down of DR5 was confirmed using WB analysis. Protein expression for FLAG in the unbound fraction was also analysed. Densitometry was performed on the DR5-DISC bound FLAG-RALB blot using ImageJ software and the results (intensity values) are denoted below the blot. Nd = not detected. Bottom: Apoptosis in the unbound fraction was determined using a Caspase-3/7 activity assay. **F** HCT116 cells were transiently transfected with EV or FLAG-RALB WT for 24 h, followed by treatment with rhTRAIL for 3 h. Expression of PARP, cleaved Caspase-3, FLAG-tag and DR5 was determined by WB (top). Apoptosis was determined using a Caspase-3/7 activity assay (bottom).

To further elucidate the mechanism by which RALB regulates rhTRAIL sensitivity, we examined the effect of DR5 agonists on RALB subcellular localisation. Fractionation experiments showed that the primary localisation for both RALB and DR5 is the membrane in untreated CRC cells (Fig. 6A). To investigate whether RALB associates with the plasma membrane-bound DR5 receptor, we used the agonistic antibody AMG 655 that binds the extracellular domain of DR5. Our experiments showed that RALB was recruited to the DISC as soon as 3 h following incubation with AMG 655 (Fig. 6B, top left and Fig. S6D). As published previously^20^, FLIP_L_ was present at the DISC exclusively in its Caspase 8-processed p43-form and FLIP_S_, FADD and Caspase-8 were also recruited. The clathrin adaptor protein 2 (AP-2) interacted with DR5 at the earliest time point indicating that endocytosis of the receptor–ligand complex is initiated at the plasma membrane upon ligand binding. Interestingly, increased proteolytic cleavage of the α subunit of AP2 (AP2α) occurred in a time-dependent manner, consistent with the results of a previous study^46^. The unbound fraction showed decreases in pro-Caspase 8 and FADD over time, consistent with increased recruitment of DISC components to the membrane in response to AMG 655 treatment (Fig. 6B, top right). Notably, silencing of RALB markedly reduced RALB recruited to the DISC, 1 h and 3 h following incubation with AMG 655 (Fig. 6B, bottom). We further investigated TRAIL-induced RALB recruitment to the DR5-DISC by immunoprecipitating FLAG-tagged RALB from untreated and TRAIL-treated cells and then immunoblotting for the presence of Myc-tagged DR5 (Fig. 6C). In agreement with the results described above, binding of RALB to DR5 occurred in TRAIL-treated cells. Furthermore, TRAIL treatment failed to recruit RALB to the DR5-DISC in Caspase-8 null cells, suggesting that the interaction between DR5 and RALB is Caspase-8-dependent (Fig. 6D).

To investigate further if RALB plasma membrane localisation and/or its GDP/GTP-bound state was required for its recruitment to the DR5-DISC in response to AMG 655 treatment, we used FLAG-tagged WT, constitutively-active (G23V), dominant-negative (S28N) and geranylgeranylation-deficient (C203S) MT RALB expression constructs (Fig. 6E). RALB C203S does not undergo geranylgeranylation at its C-terminal domain and is localised in the cytosol fraction^47^. Binding of RALB to the DR5-DISC was attenuated by the C203S-MT but not the S28N-MT, when compared to WT-RALB in AMG 655-treated cells (Fig. 6E). Interestingly, a decreased association of G23V-RALB with the DR5-DISC was observed compared to WT or S28N-RALB, suggesting that constitutively active RALB has a higher turnover rate and may be removed from the plasma membrane more rapidly, compared to WT-RALB. Notably, transient overexpression of WT-RALB attenuated TRAIL-induced apoptosis in HCT116 CRC cells (Fig. 6F). AMG 655-induced apoptosis was unaffected and slightly increased following transient overexpression of G23V-RALB, S28N-RALB and C203S-RALB MT expression constructs, respectively (Fig. 6E, lower panel). Taken together, these results are the first to our knowledge to demonstrate the importance of RALB in regulating sensitivity to TRAIL treatment in *KRAS*MT CRC.

## Discussion

Identification of novel therapeutic strategies that can induce effective *KRAS*MT-tumour-cell killing remains an unmet need^1^. So far, all attempts to interfere with oncogenic RAS signalling have failed in the clinic. In particular, combinations of inhibitors of the MAPK and PI3K/Akt RAS effector signalling have been shown to be ineffective in *KRAS*MT CRC^4^, suggesting the involvement of additional effector pathways in mediating *KRAS* oncogenic signalling. In this study, we provide evidence that the RALB-GTPase is an important regulator of *RAS*MT cancer cell survival and that inhibition of RALB ‘primes’ *KRAS*MT cells to death upon stimulation with DR5-agonistic antibodies.

Previous studies have shown that RAL-GEF pathway activation is one of the basic requirements for RAS-mediated transformation of human epithelial cells^7,8^ and that RALA and RALB have both distinct and/or overlapping roles in regulating migration/invasion and anchorage-independent growth in pancreatic, bladder and prostate cancer^11,48^. In agreement with a previous study in a range of cancer cell models^49^, we found that RALB, and *not* RALA, has a profound effect on the survival and apoptosis of CRC cells, in particular in *KRAS*MT CRC. Contrary to a previous study from Martin et al.^50^, our study showed that overexpression of RALB markedly increased the colony-forming ability of *KRAS*MT CRC cells. Our previous studies and those of others have shown that MEK1/2 inhibition is relatively ineffective at inducing apoptosis in *KRAS*MT CRC cells^15^. An important finding of our study is that neither the RALB nor the RALA RAS effector pathways are involved in regulating resistance to MEK1/2 inhibition in *KRAS*MT CRC. Taken together, we demonstrate here for the first time a key role for RALB as a regulator of survival in *KRAS*MT CRC.

High RALB expression has been reported in several tumours, including lung cancer, and has been identified as a poor prognostic marker in these tumours^51^. In this study, we found that RALB was overexpressed in CRC tissues compared to matched normal colon tissue, highlighting the potential for exploitation of RALB as a therapeutic target in CRC. Stage II/III CRC patients with high *RALB* expression trended towards poorer overall survival but the data were not statistically significant. Importantly, further analysis revealed that the highest levels of *RALB* are detected in CRIS-B CRC, the subgroup that is characterised by invasive, epithelial-to-mesenchymal transition features and strong TGF-β activity, which is associated with a worse outcome^13^. Previous studies have also shown a positive correlation between RALB and an invasive, aggressive and metastatic phenotype in bladder and pancreatic cancer^10,52^. These results would indicate that anti-RALB selective therapies may provide an effective therapeutic approach for *KRAS*MT CRIS-B CRC with high expression levels of RALB.

Several studies have shown that downregulation of RALB results in apoptosis and increased drug sensitivity through de-phosphorylation of TBK1, resulting in decreased expression of NF-κB target pro-survival genes (e.g. Bcl-2)^53,54^. In contrast to these studies, our data did not show a role for TBK1 in mediating siRALB-induced apoptosis (Fig. S6E). However, the mechanism by which RALB silencing induces apoptosis was shown to involve Caspase-8-dependent apoptosis following upregulation of cell surface and total DR5 levels. This is the first demonstration of a link between RALB and DR5 expression. Moreover, using receptor-specific siRNA and a DR5 CRISPR knockout model, we further found that induction of apoptosis following siRALB was mediated by DR5, but not DR4.

It was previously shown that DR5 cell-surface levels are a critical mediator to TRAIL-induced apoptosis^46,55^. Consistent with this newly identified association between RALB and DR5, we have shown that inhibition of RALB through specific siRNA or the broad spectrum cyclin-dependent kinase inhibitor Dinaciclib significantly sensitised *KRAS*MT CRC cells to TRAIL treatment. We also found that transiently overexpressing RALB decreased DR5 levels and apoptosis in response to TRAIL treatment. Of note, this is also the first study showing that stimulation of DR5 results in a rapid and Caspase-8-dependent association of RALB with the DR5-DISC, which only occurred when RALB was able to translocate to the plasma membrane. Several clinical trials using fully human DR5-agonistic antibodies, including AMG 655, have been unable to show meaningful clinical efficacy^56^, suggesting inherent resistance of primary tumours to DR5 activation-induced apoptosis. Our finding indicates a novel and effective way to enhance DR5 activation-induced apoptosis, in particular for *KRAS*MT CRC.

Given its potential role in resistance to DR5-agonistic agents, we further investigated how DR5 expression levels are regulated by RALB. This is the first study showing that RALB regulates DR5 protein levels through the lysosomal degradation pathway. A number of reports have shown that DR5 levels are controlled by the lysosomal, but not the proteasomal degradation pathway^42,57^. Additional studies have shown that DR5 co-localises and interacts with LC3-II in autophagosomes, in particular in TRAIL-resistant cells^55^. These reports show that lysosomal inhibition increases cell-surface DR5 levels, as knockdown of RALB did in our study (Fig. 3A, B). Using transient overexpression of RALB, we found that DR5 co-localises with lysosomes in *KRAS*MT CRC cells. These results would indicate that anti-RALB selective therapies may suppress DR5 lysosomal degradation, increasing cell membrane DR5 levels and thereby augmenting sensitivity to DR5-agonistic antibodies.

In conclusion, we have shown that RALB inhibition regulates DR5 dynamics and induces apoptotic priming, and that targeting this apoptotic vulnerability with selective DR5-agonistic antibodies may be a promising strategy to improve treatment response in *KRAS*MT CRC tumours. Furthermore, the study has uncovered a novel RALB-DR5-DISC complex with RALB overexpression suppressing TRAIL-induced apoptosis. Importantly, RALB is highly expressed in the poor prognostic CRIS-B CRC subgroup. From a clinical perspective, our data provides the preclinical rationale for the initiation of a phase I study of RALB inhibition with novel multivalent TRAIL receptor 2/DR5 agonists (eg. MEDI3039)^58,59^ in *KRAS*MT CRIS-B CRC with high expression levels of RALB. Our findings also support the development of new and specific anti-RALB molecules that may provide tools to investigate the biology and therapeutic potential of RALB in CRC as they become available^21^.

## Supplementary information

Supplementary figure 1

Supplementary figure 2

Supplementary figure 3

Supplementary figure 4

Supplementary figure 5

Supplementary figure 6

Supplementary table 1

Supplementary figure legends
