# Supplementary figures and images for: RALB GTPase: a critical regulator of DR5 expression and TRAIL sensitivity in KRAS mutant colorectal cancer
Source: Cell Death Dis. 2020 Oct 29;11(10):930. doi: 10.1038/s41419-020-03131-3 (PMC7596570; doi:10.1038/s41419-020-03131-3)

Supplementary Figure 1

A.

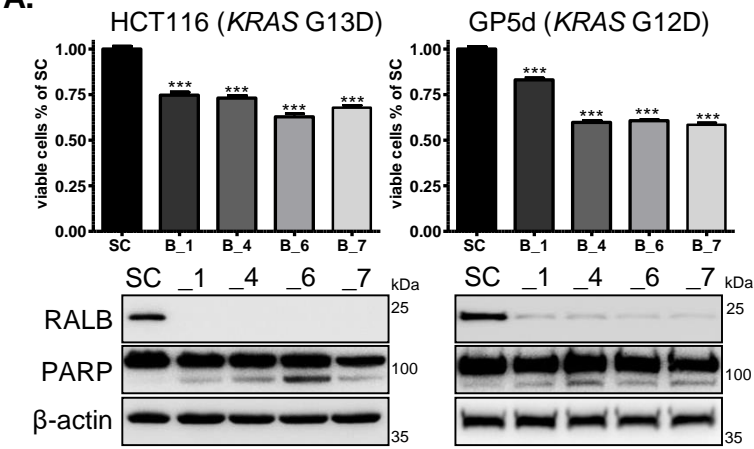

B.

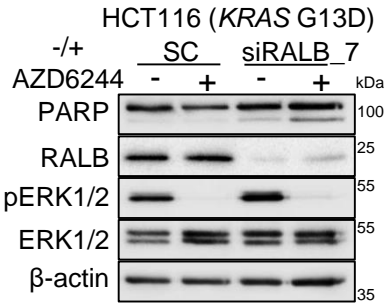

C.

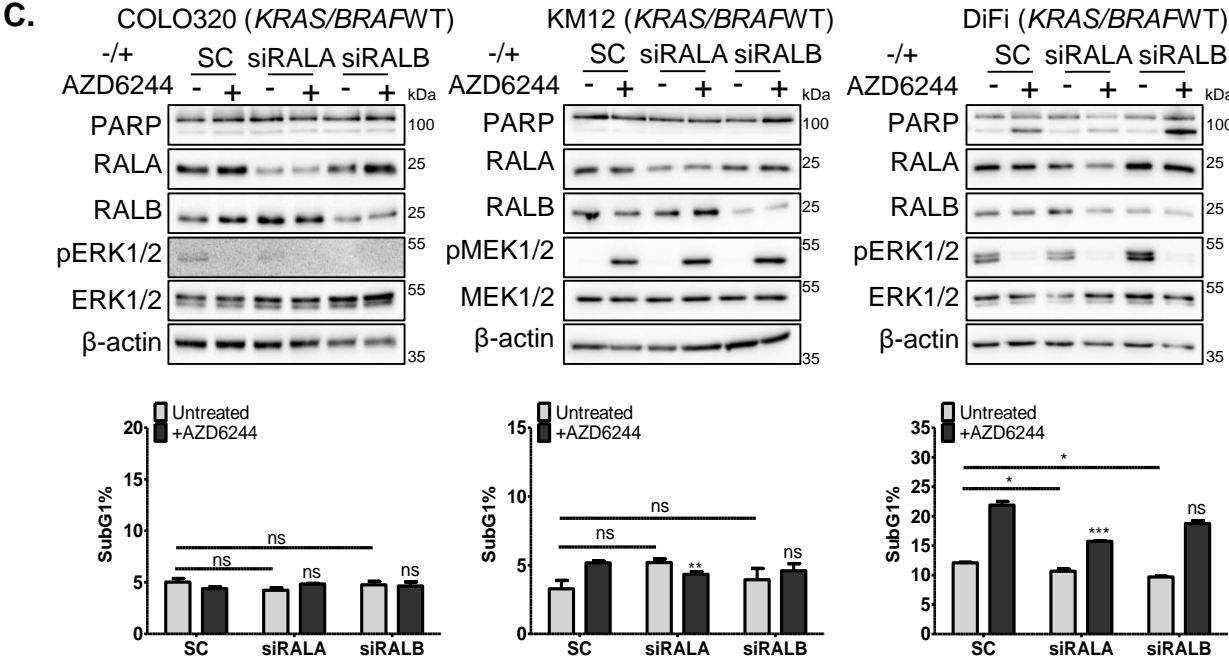

D.

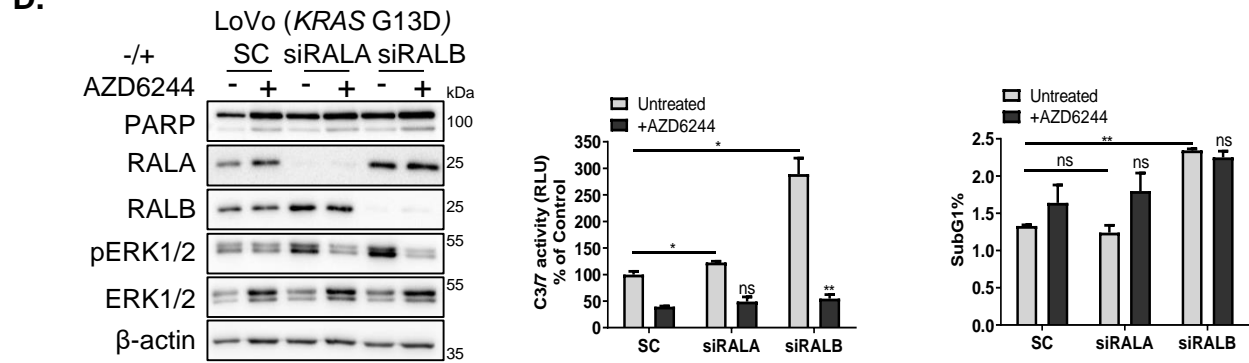

Supplement: Supplementary file 1 — Supplementary figure 1 [file 41419_2020_3131_MOESM1_ESM.pdf]

Supplementary Figure 2

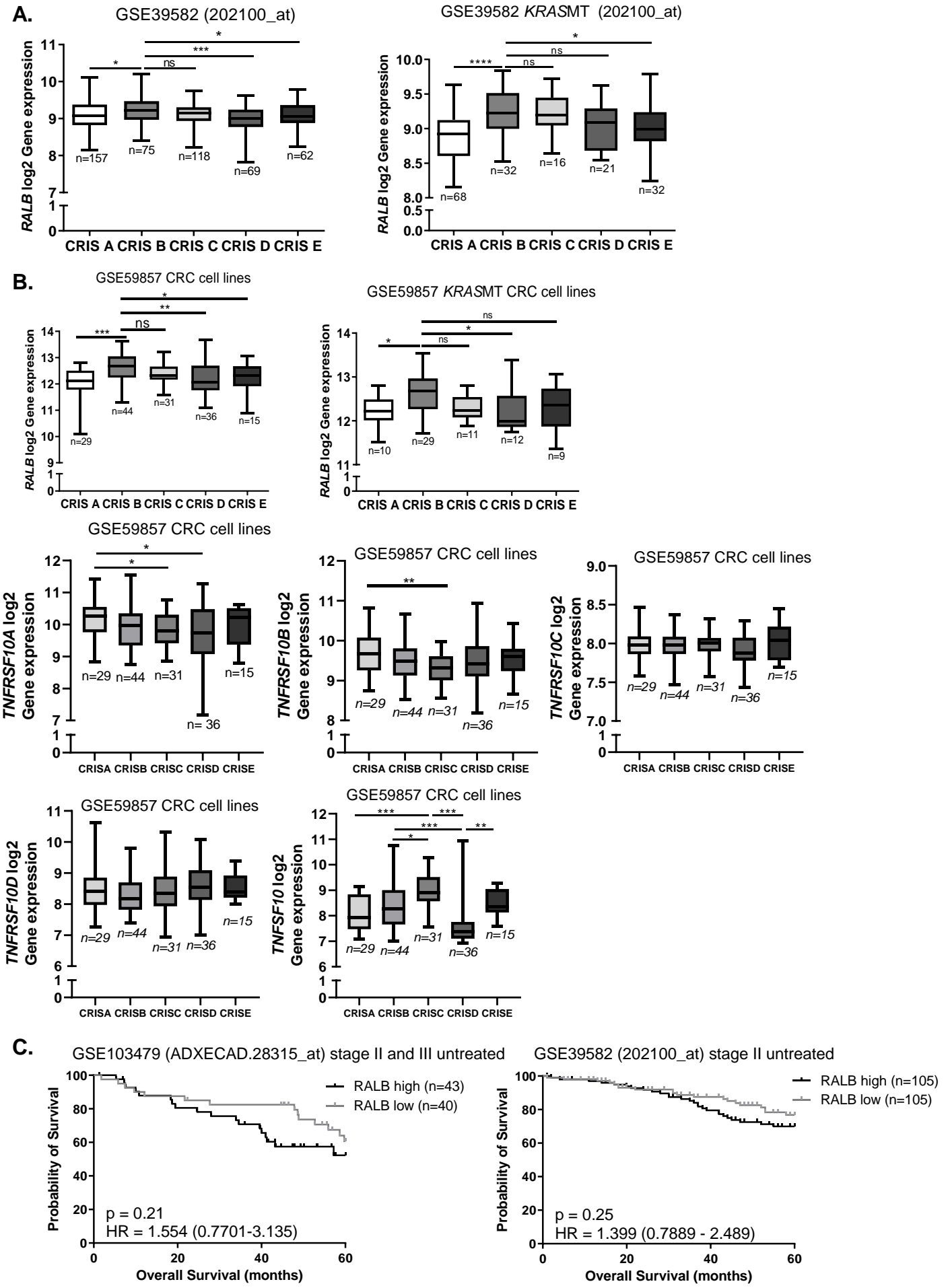

D.

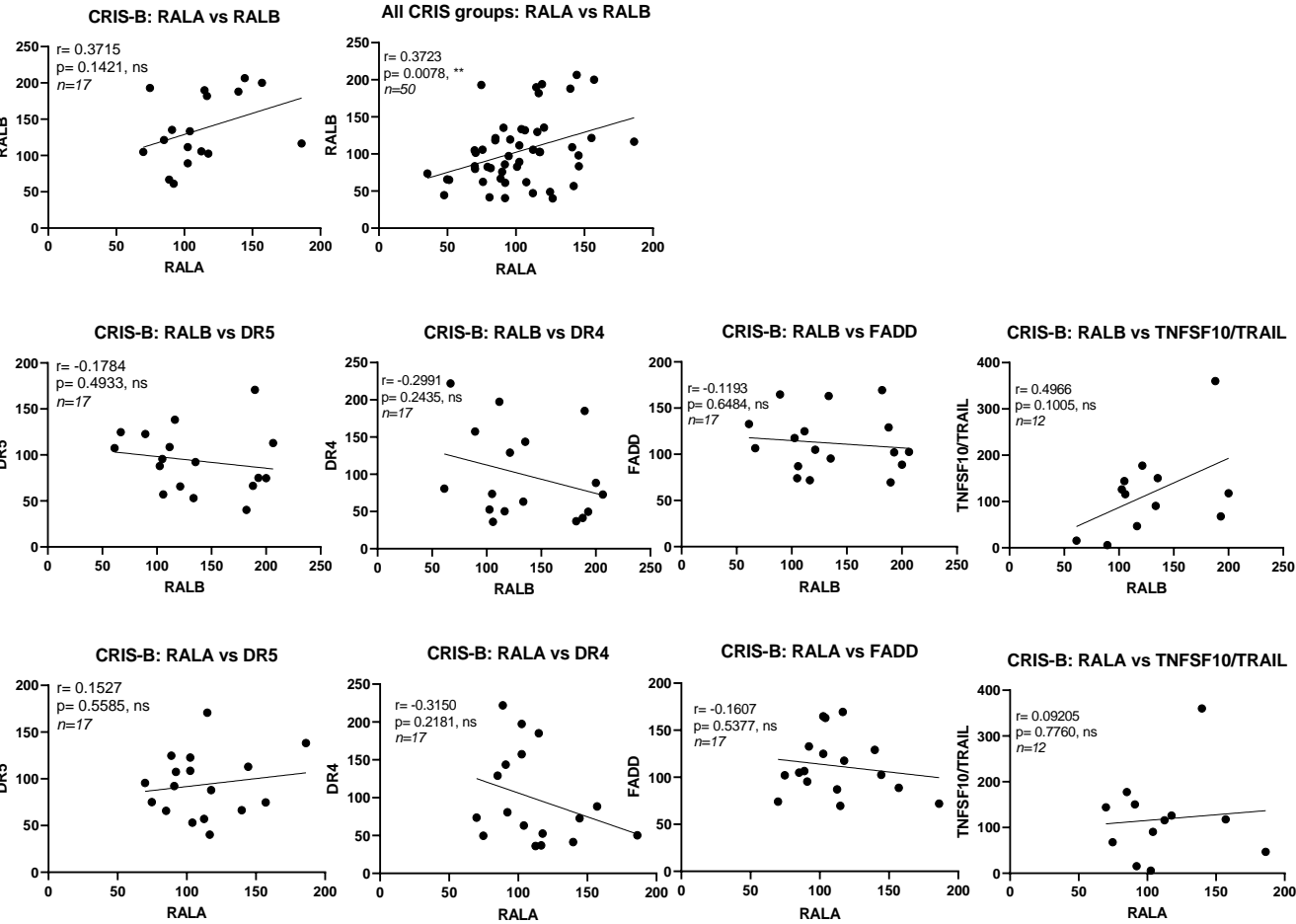

E.

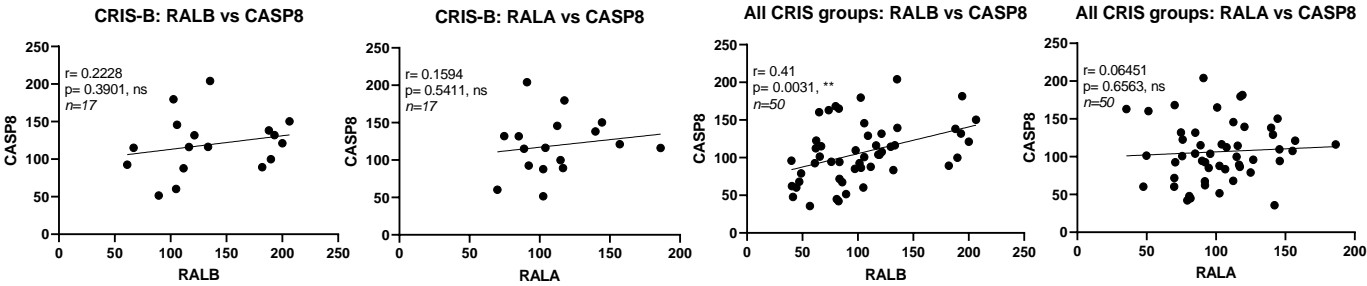

F.

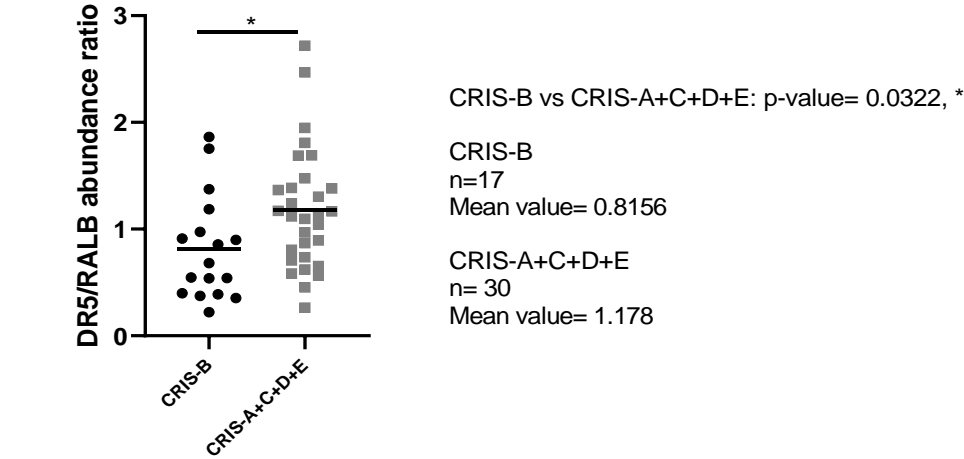

Supplement: Supplementary file 2 — Supplementary figure 2 [file 41419_2020_3131_MOESM2_ESM.pdf]

Supplementary Figure 3

A.

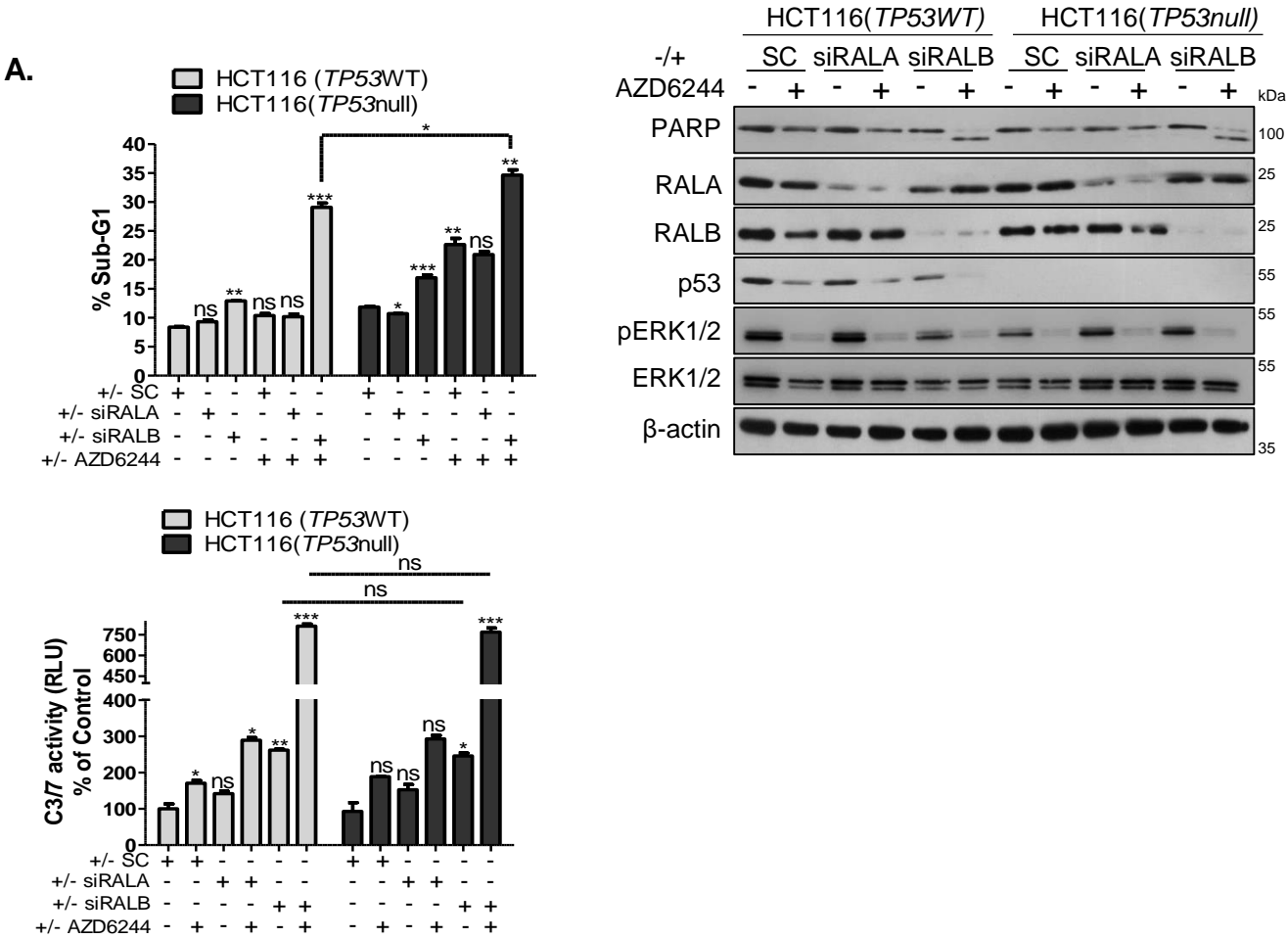

B.

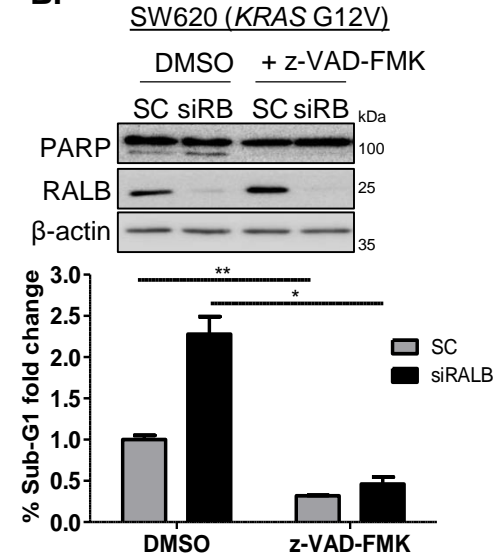

C.

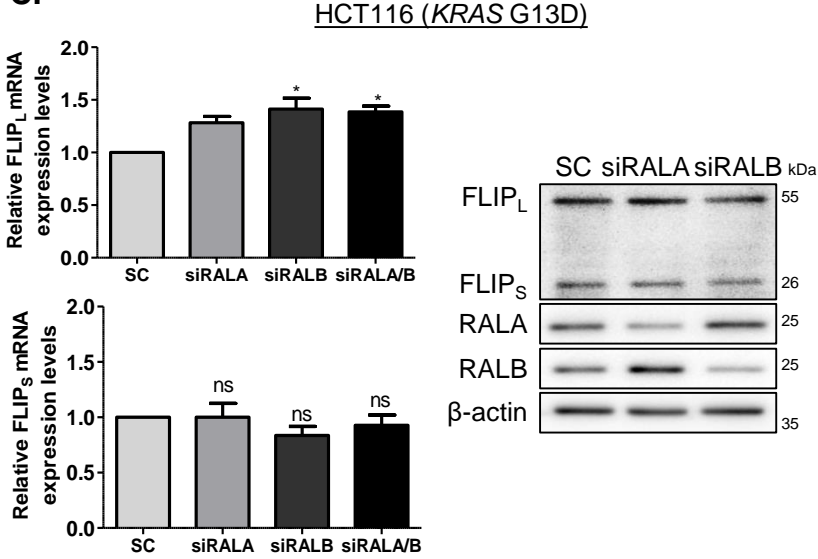

Supplement: Supplementary file 3 — Supplementary figure 3 [file 41419_2020_3131_MOESM3_ESM.pdf]

Supplementary Figure 4

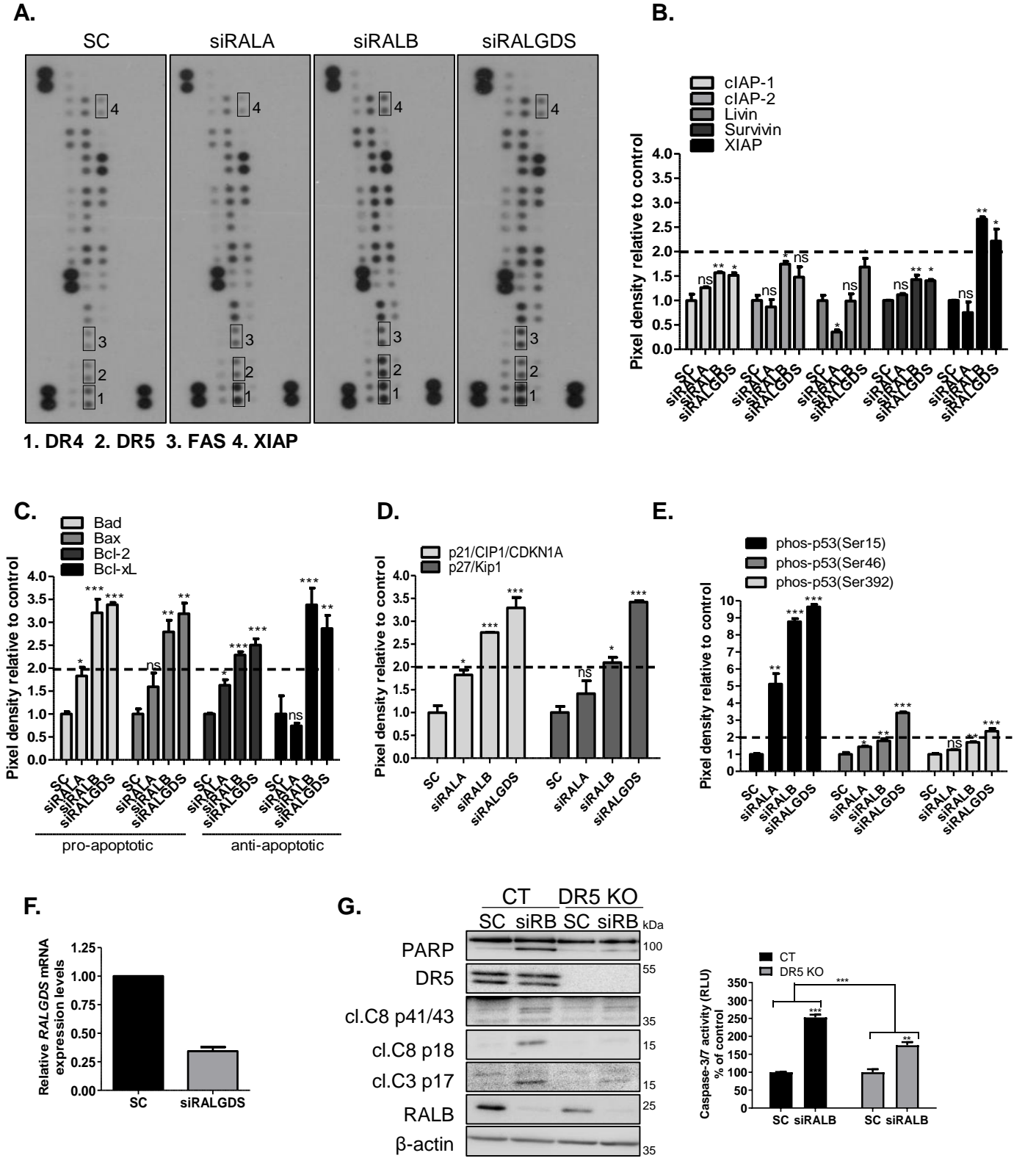

Supplement: Supplementary file 4 — Supplementary figure 4 [file 41419_2020_3131_MOESM4_ESM.pdf]

**Supplementary Figure 5**

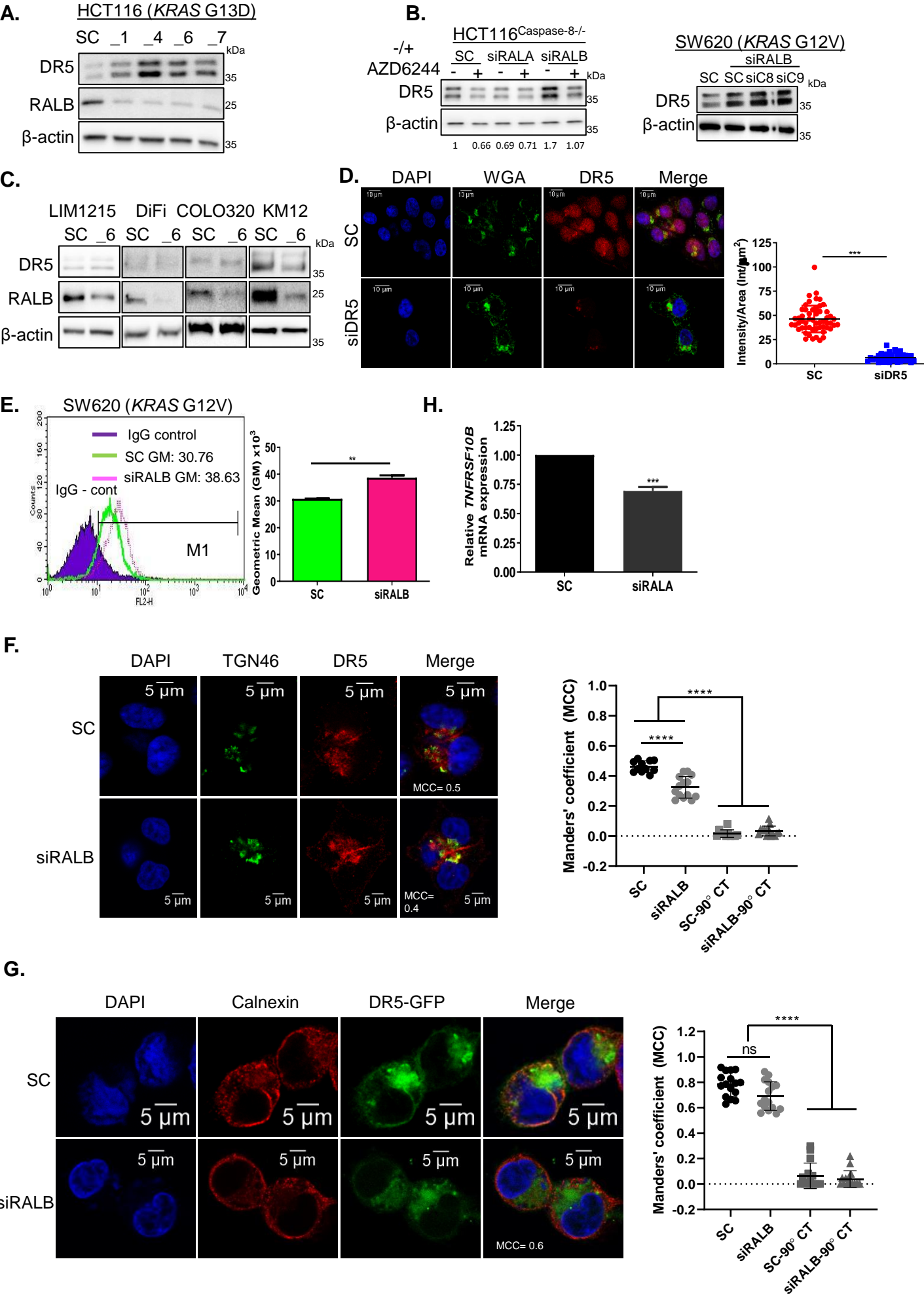

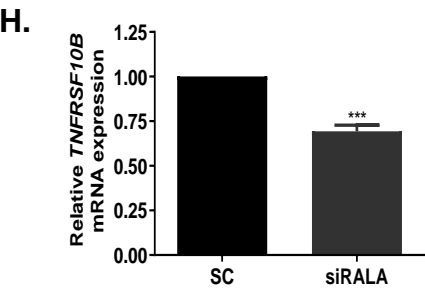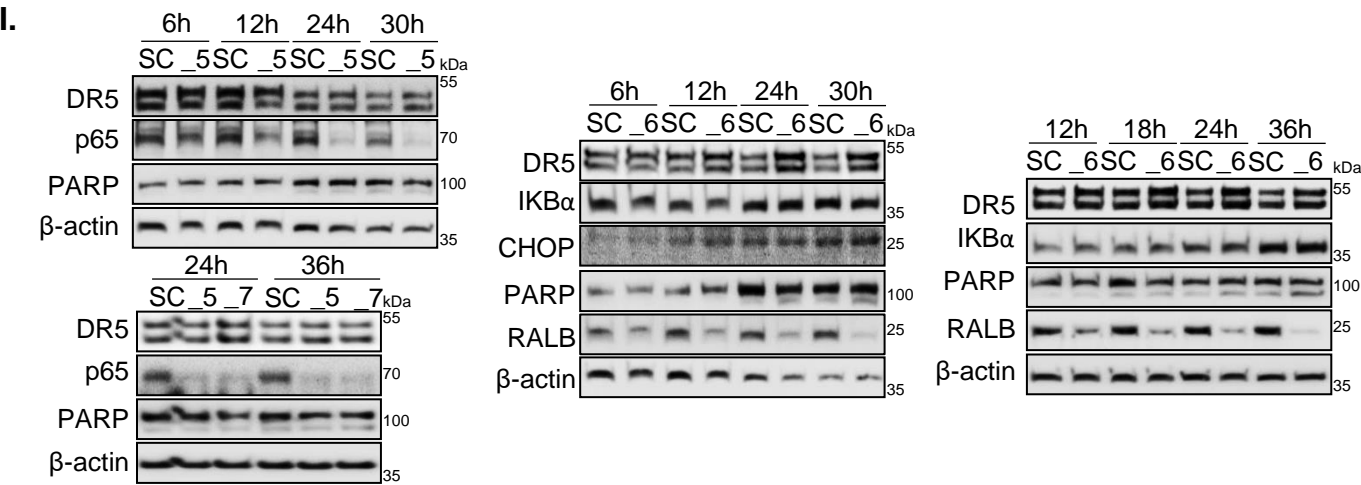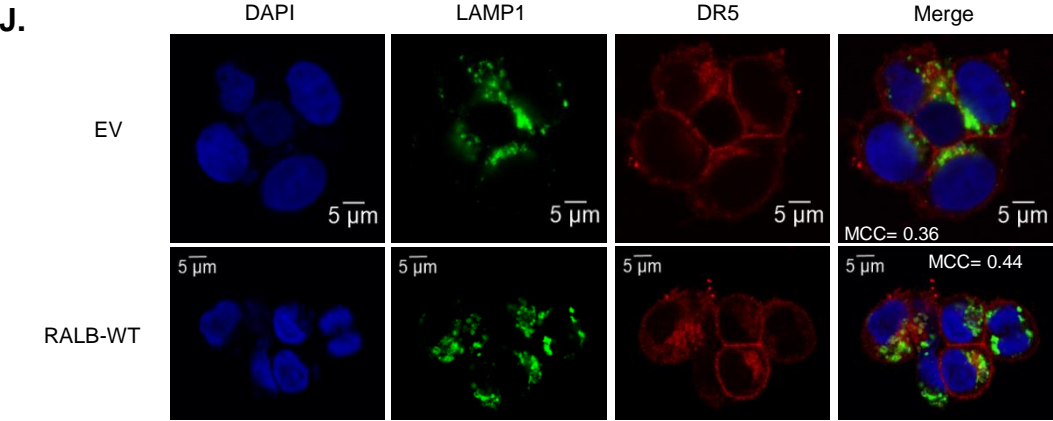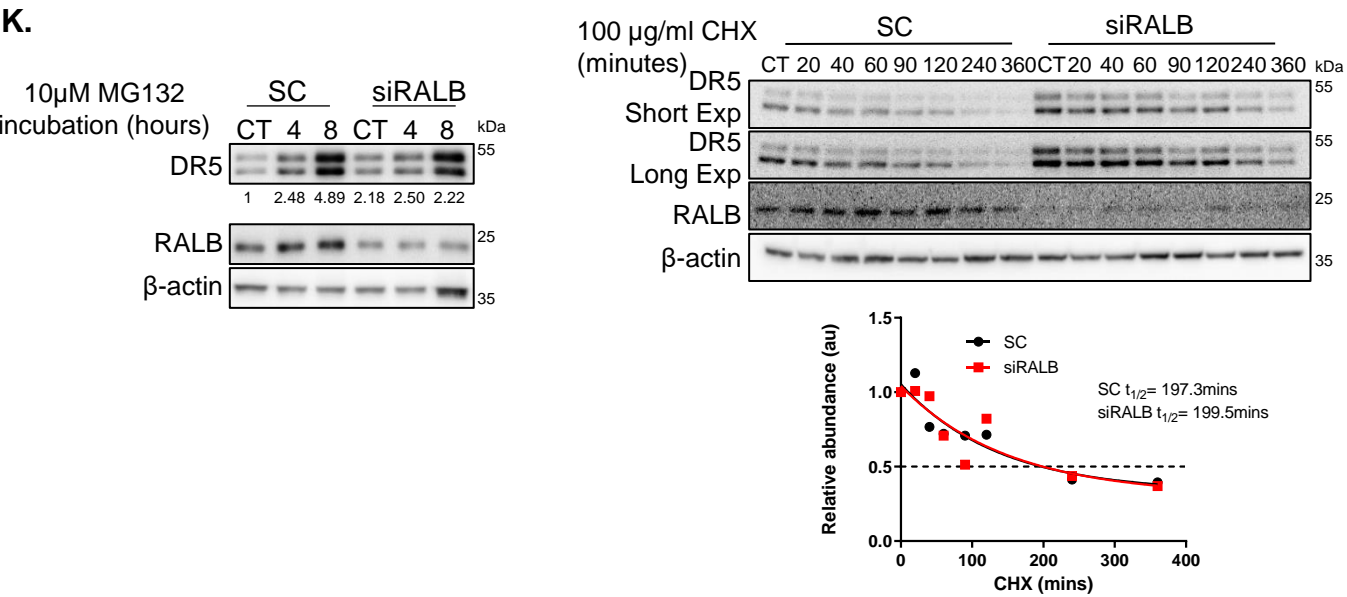

Supplement: Supplementary file 5 — Supplementary figure 5 [file 41419_2020_3131_MOESM5_ESM.pdf]

Supplementary Figure 6

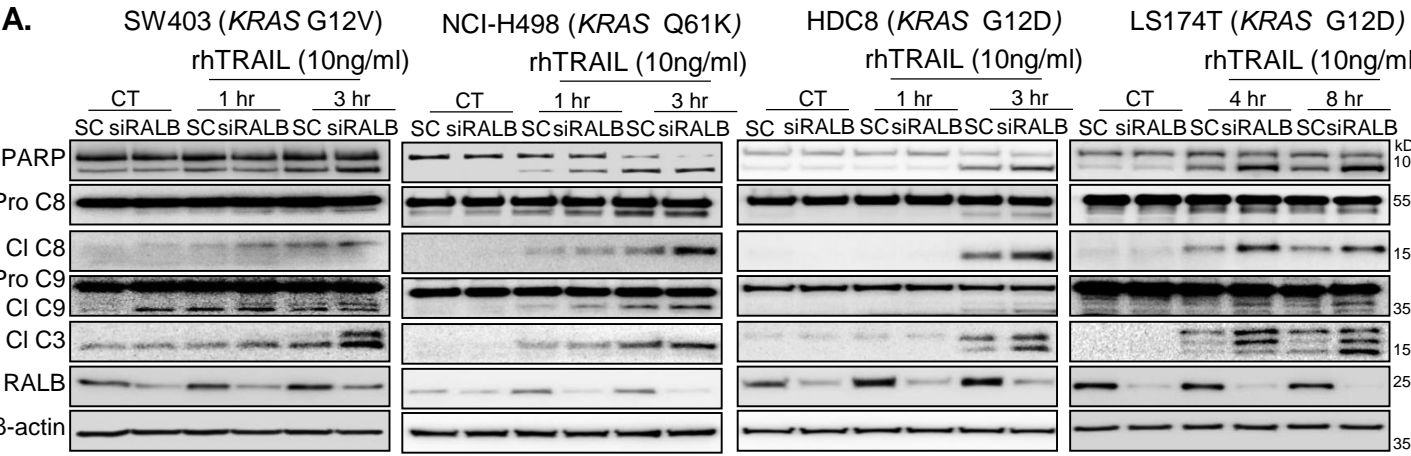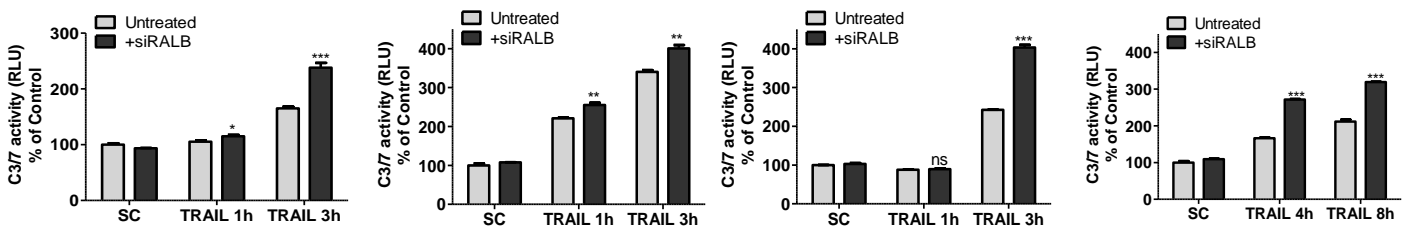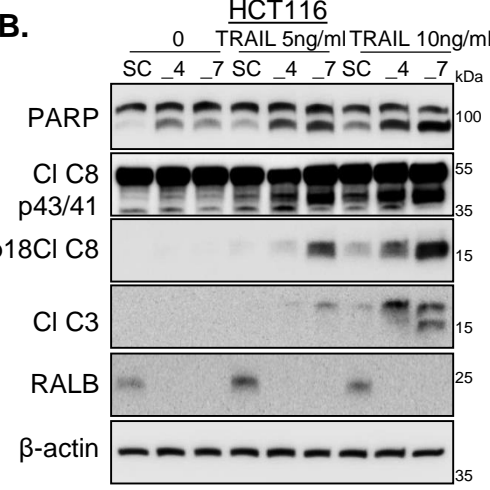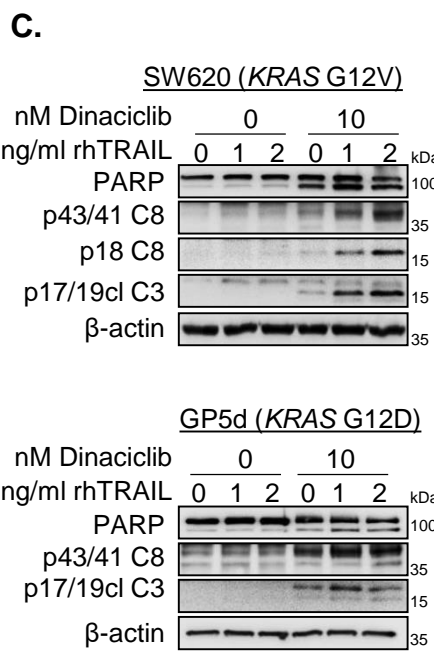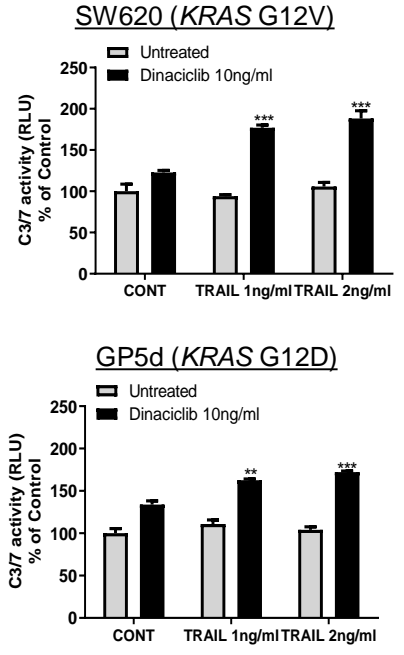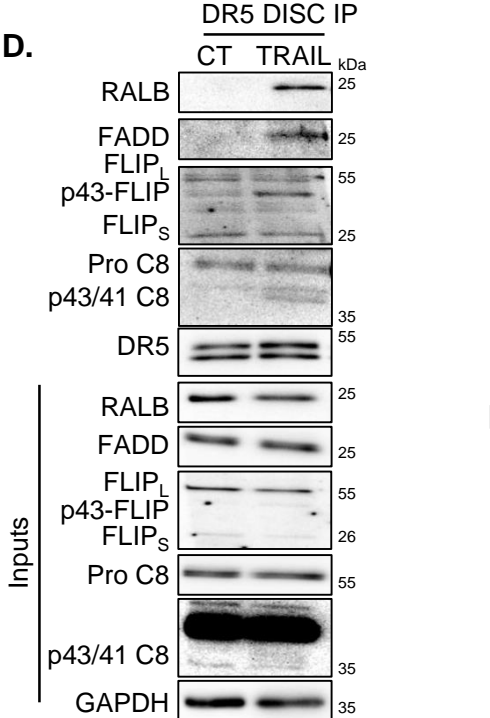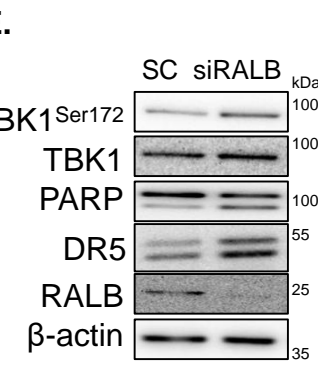

Supplement: Supplementary file 6 — Supplementary figure 6 [file 41419_2020_3131_MOESM6_ESM.pdf]
