# Supplementary material for: RALB GTPase: a critical regulator of DR5 expression and TRAIL sensitivity in KRAS mutant colorectal cancer
Source: Cell Death Dis. 2020 Oct 29;11(10):930. doi: 10.1038/s41419-020-03131-3 (PMC7596570; doi:10.1038/s41419-020-03131-3)
Supplement: Supplementary file 7 — Supplementary table 1 [file 41419_2020_3131_MOESM7_ESM.pdf]

Supplementary Table 1

Antibody	Supplier	Product code	Application
Anti-human CD262 (DR5) PE	ThermoFisher Scientific	12-9908-42	Flow cytometry
Anti-mouse HRP	Cell Signalling Technology	7076	Western blot
Anti-Rabbit HRP	Cell Signalling Technology	7074	Western blot
AP2α	Abcam	ab2730	Western blot
Bcl-xL (54H6)	Cell Signalling Technology	2764	Western blot
Calnexin	Abcam	22595	Immunofluorescence
Caspase-8 (12F5)	Enzo	ALX-804-242-C100	Western blot
Caspase-9	Cell Signalling Technology	9502	Western blot
CHOP	Cell Signalling Technology	2895	Western blotting
Cleaved Caspase-3	Cell Signalling Technology	9661	Western blot
DR4 (D9S1R)	Cell Signalling Technology	42533	Western blot
DR5 (D4E9) XP®	Cell Signalling Technology	8074	Western blot, Immunofluorescence
EGFR	BD Biosciences	610017	Western blot
ERK1/2	Cell Signalling Technology	9102	Western blot
FADD (A66-2)	BD Biosciences	556402	Western blot
FLAG (M2) HRP	Sigma	A8592	Western blot
FLIP (NF6)	Cal tag medsystems	AG-20B-0056-C100	Western blot
GAPDH	Novus Biologicals	NB300-221	Western blot
IKBα	Cell Signalling Technology	9242	Western blotting
LAMP1	Abcam	ab25630	Immunofluorescence
LC3-A/B	Cell Signalling Technology	12741	Western blot
Mcl-1	BD Transduction	559027	Western blot
MEK1/2	Cell Signalling Technology	9122	Western blot
Mouse IgG1 kappa isotype control, PE	ThermoFisher Scientific	12-4714-41	Flow cytometry
Myc-Tag (71D10)	Cell Signalling Technology	2278	Western blot
p53 (DO-1)	Santa Cruz	sc-126	Western blot
p65	Santa Cruz	sc-8008	Western blot
PARP	Cell Signalling Technology	9542	Western blot
pERK1/2 (Thr202/Tyr204)	Cell Signalling Technology	9101	Western blot
pMEK1/2 (Ser217/221)	Cell Signalling Technology	9154	Western blot
pTBK1 (Ser172)	Cell Signalling Technology	5483	Western blot
RALA	BD Transduction	610221	Western blot
RALB	Cell Signalling Technology	3523	Western blot
TBK1	Cell Signalling Technology	3504	Western blot
TGN46	Bio-Rad	AHP500GT	Immunofluorescence
XIAP	Cell Signalling Technology	2042	Western blot
β-actin (AC-74)	Sigma	A2228	Western blot
