# Supplementary material for: RALB GTPase: a critical regulator of DR5 expression and TRAIL sensitivity in KRAS mutant colorectal cancer
Source: Cell Death Dis. 2020 Oct 29;11(10):930. doi: 10.1038/s41419-020-03131-3 (PMC7596570; doi:10.1038/s41419-020-03131-3)
Supplement: Supplementary file 8 — Supplementary figure legends [file 41419_2020_3131_MOESM8_ESM.pdf]

Supplementary Figure 1

Supplementary Figure 1. Role of RALB in survival of *RAS*MT and *RAS*WT CRC cells. A. Upper: Crystal Violet cell viability assays in *KRAS*MT HCT116 and GP5d cells following transfection with 10nM of SC or 4 different siRNA sequences (sequence _1, _4, _6 and _7) against RALB for 72h. The percentage of viable cells relative to SC are plotted. **Lower:** Western blotting (WB) for RALB and PARP following transfection with 10nM of SC or 4 different siRNA sequences against RALB for 72h. **B.** HCT116 cells were transfected with 10nM SC or siRALB_7 and co-treated with AZD6244 for 24h and PARP, RALB, pERK1/2$^{T202/Y204}$ and ERK1/2 determined by WB. **C. Upper:** *RAS*WT CRC cells were transfected with 10nM SC, siRALA or siRALB and co-treated with 1μM AZD6244 for 24h and PARP, RALA, RALB, pERK1/2 or pMEK1/2$^{S217/221}$, ERK1/2 or MEK1/2 determined by WB. **Lower:** PI Flow cytometric analysis of *RAS*WT cells, transfected with 10nM SC, siRALA or siRALB and co-treated with 1μM AZD6244 for 48h. **D. Left:** LoVo cells were transfected with 10nM SC, siRALA or siRALB and co-treated with 1μM AZD6244 for 24h and PARP, RALA, RALB, pERK1/2, ERK1/2 determined by WB. **Middle:** Caspase-3/7 activity was determined using the Caspase-Glo® 3/7 kit (Promega) on cell lysates. **Right:** PI Flow cytometric analysis of LoVo cells, transfected with 10nM SC, siRALA or siRALB and co-treated with 1μM AZD6244 for 48h. For all western blots β-actin was used as a loading control.

Supplementary Figure 2

Supplementary Figure 2. Clinical significance of RALB in CRC. A. Boxplots representing the log2 gene expression values for *RALB* across CRIS subgroups in the entire GSE39582 clinical dataset (**left**) and the GSE39582 *KRAS*MT clinical dataset (**right**). **B. Top:** Boxplots representing the log2 gene expression values for *RALB* across CRIS subgroups in the entire GSE59857 CRC cell line dataset (**left**) and the GSE59857 *KRAS*MT CRC cell line dataset (**right**). **Middle and Bottom:** Boxplots representing the log2 gene expression values for *TNFRSF10A, TNFRSF10B, TNFRSF10C, TNFRSF10D* and *TNFSF10* across CRIS subgroups in the entire GSE59857 CRC cell line dataset. A one-way ANOVA with multiple comparisons analysis was performed to compare CRIS-B to the other CRIS-groups. 'N' numbers below the boxplots indicate the sample numbers for each group within the dataset. **C.** Kaplan-Meier analysis, using the log-rank (Mantel-Cox) statistical test of CRC GSE103479 dataset (probe ID: ADXECAD.28315_at) and GSE39582 (probe ID: 202100_at) for overall survival in stage II/III CRC patients. Survival curves and p-values for RALB low and RALB high are presented. 'N' refers to the sample number in the RALB high and low groups. Patients alive after 60 months was considered censored. **D&E.** Correlation analyses for the indicated proteins across the specified CRIS groups was performed in Prism using the COREAD cell line dataset. Linear regression analysis was performed in Prism to produce a line on the graphs presented. 'N' indicates the number of cell lines used for the analyses. **F.** The ratio of DR5 to RALB abundance in CRIS-B cell lines versus CRIS-A, C, D and E cell lines was calculated using the COREAD dataset. An unpaired t-test was used to compare the two groups. Data was graphed and analysed using Prism. Horizontal lines on the graph represent mean values for both groups.

Supplementary Figure 3

Supplementary Figure 3. Role of p53 and caspases in regulating apoptosis following siRALB in *RAS*MT CRC. A. HCT116 p53 wild type (*TP53*WT) cells and its isogenic p53 null (*TP53*null) clone were transfected with 10nM SC, siRALA or siRALB and co-treated with 1μM AZD6244 for 48h and apoptosis was assessed by flow cytometric analysis with Propidium Iodide **(Upper, left),** Western blotting for PARP **(Upper, right)** or Caspase-3/7 activity **(Lower, left)**. **B.** SW620 cells were transfected with 10nM SC or siRALB and pre-incubated with DMSO or 20 μM of the pan-caspase inhibitor. Apoptosis was determined by PARP (**upper**) and flow cytometric analysis with Propidium Iodide (**lower**). **C. Left:** HCT116 cells were transfected with 10nM SC, 10nM siRALA, 10nM siRALB or 10nM siRALA and 10nM siRALB for 24h and $FLIP_L$ and $FLIP_S$ mRNA expression levels determined by Real-time PCR. Relative mRNA expression was calculated using the ΔΔCt method with normalisation to *ACTB* and *GAPDH* performed**.** mRNA expression is presented relative to SC control. **Right:** HCT116 cells were transfected with 10nM SC, siRALA or siRALB for 24h. $FLIP_L$, $FLIP_S$, RALA and RALB expression levels were determined by WB. β-actin was used as a loading control for all WB analysis.

Supplementary Figure 4

Supplementary Figure 4. Impact of RALB depletion on expression of pro-apoptotic and anti-apoptotic proteins. A. Human apoptosis array in HCT116 cells, 24h following transfection with 10nM SC, siRALA, siRALB or siRALGDS. Boxes and numbers indicate results for DR4, DR5, FAS and XIAP. **B, C, D** and **E**: Densitometry was performed on the array panels using ImageJ software. Expression values were normalized to respective scrambled (SC) control values, data is presented as pixel density relative to control. Data for IAPs (Inhibitors of apoptosis) (**B**), Bcl-2 family members (**C**), cell cycle regulatory proteins, p21 and p27 (**D**) and phosphorylated p53 (**E**) are shown. The dashed line represents a fold-change of 2. **F.** HCT116 cells were transfected with 10nM SC or siRALGDS for 24h. *RALGDS* mRNA was quantified using RT-PCR. Raw values were normalised to expression of housekeeping genes *ACTB* and *GAPDH* and were analysed using the ΔΔCT method. mRNA values are presented relative to SC control. **G**. Paired CRISPR parental (control=CT) and DR5 knockout (KO) cells were transfected with 10nM SC or RALB for 48h. Apoptosis was determined by WB for PARP, Caspase-8 and Caspase-3 (**left**), and Caspase-3/7 activity (**right**).

Supplementary Figure 5

Supplementary Figure 5. RALB depletion upregulates DR5 in *RAS*MT CRC cells. A. HCT116 cells were transfected with 10nM SC or RALB (multiple sequences_1, _4, _6, _7) for 24h. Cells were harvested and protein expression of DR5 and RALB determined using WB. **B. Left:** HCT116$^{Cspase\text{-}8\text{-}/\text{-}}$ cells were transfected with 10nM SC, RALA or RALB and co-treated with 1µM AZD6244 for 24h. DR5 levels were determined by WB. **Right:** CRC cells were transfected with 10nM C8 or C9 siRNA for 24h and thereafter transfected with 10nM RALB siRNA for 24h and DR5 levels assessed by WB. **C.** *RAS*WT LIM1215, DiFi, COLO320 and KM12 were transfected with 10nM SC or RALB (_6) for 24h and DR5 expression was determined by WB. **D. Left:** HCT116 CRC cells were seeded on coverslips in duplicate and were transfected with 10nM SC or DR5 for 48h. Cells were fixed in 4% Paraformaldehyde (PFA) and were stained with Alexa Fluor 488-conjugated wheat germ agglutinin (WGA, green), followed by permeabilisation and counterstaining with DR5 antibody (red) and DAPI. Images were obtained with a SP8 confocal microscope. **Right:** >20 cells were scored for fluorescence intensity and compared to their respective controls. Analysis is based on one experiment. **E.** SW620 cells were transfected with 10nM SC or siRALB for 24h and DR5 cell membrane expression was assessed by flow cytometry using a receptor-specific PE-conjugated mAb. Geometric mean (fluorescence intensity) of DR5 cell surface expression was compared with a matched-isotype control antibody. **F&G.** Impact of RALB depletion on colocalisation between DR5 and the Golgi marker, TGN46, and the Endoplasmic Reticulum marker, Calnexin. **F.** HCT116 cells were transfected with siRNA targeted against either SC or RALB for 24h, following which cells were fixed, permeabilised and blocked overnight. Cells were then stained with DR5 and TGN46 antibodies, followed by incubation with mounting medium and DAPI. Coverslips were added and sealed. Images were collected using an SP8 confocal microscope with fixed laser settings between different treatment groups. Manders' colocalisation coefficient (MCC) was used to calculate the fraction of total DR5 fluorescence (red) overlapping TGN46 fluorescence (green) in ImageJ. As a negative control, TGN46 images were rotated 90° and MCC was re-calculated to rule out random colocalisation. Over 60 cells were analysed per treatment group across 2 independent experiments. Data is representative of both experiments, with error bars indicating standard deviation from the mean. A one-way ANOVA with multiple comparisons test was performed to compare the different groups. **G.** HCT116 cells were transfected with GFP-DR5 and treated with siRNA targeting SC or RALB for 24h. Cells were stained with an antibody for the ER marker, Calnexin following fixation and were prepared for microscopy as above. Images were taken as described above. MCC was calculated to measure the fraction of total GFP-DR5 overlapping with Calnexin staining, with 90° negative controls employed by rotating GFP-DR5 as explained in **F**. Over 60 cells were analysed per treatment group across 2 independent experiments. Data is representative of both experiments, with error bars indicating standard deviation from the mean. A Kruskal Wallis one-way ANOVA with multiple comparisons test was performed in Prism to compare the different groups. MCC values are presented rounded up to 1 decimal place in white font on the respective 'Merge' images. **H.** HCT116 cells were transfected with 10nM SC or siRALA. Transcriptional expression of DR5 (*TNFRSF10B*) was determined using RT-PCR and is presented relative to SC. Raw values were normalised to expression of housekeeping genes *ACTB* and *GAPDH* and were analysed using the ΔΔCT method. **I. Left:** HCT116 cells were transfected with 10nM SC or siRELA (sip65; denoted by sequences_5 and 7) for the indicated time and protein expression of DR5, p65, PARP and β-actin determined using WB. **Middle** and **Right:** HCT116 cells were transfected with 10nM SC or siRALB (_6) for the indicated time and expression of IKBα, RALB, DR5, CHOP, PARP and β-actin was determined using WB. **J.** HCT116 cells were transfected with either EV or FLAG-RALB WT for 24h. Cells were fixed, permeabilised, blocked, and then stained with a lysosomal marker LAMP1, and DR5 antibody. Manders' colocalisation coefficient (MCC) was calculated using ImageJ to measure the fraction of total DR5 fluorescence overlapping with LAMP1 fluorescence for each treatment and is presented in the 'Merge' image rounded to 2 decimal places. Image presented is representative of three independent experiments. **K. Left panel:** HCT116 cells were treated with siRNA targeting either SC or siRALB for a total transfection time of 24 hours. Cells were treated with 10µM MG132 for 4 or 8 hours. The expression of DR5, RALB and β-actin was analysed using WB. The numbers at the bottom of the WB panel represent densitometry performed on the DR5 blot using ImageJ (DR5 densitometry values were normalised to β-actin). **Right panel:** HCT116 cells were treated with siRNA targeting either SC or siRALB for a total transfection time of 24 hours. Cells were treated with 100 µg/ml CHX (Cycloheximide) for the indicated times. The expression of DR5, RALB and β-actin was analysed using WB. Densitometry was performed on the DR5 blot using ImageJ. Raw densitometry values were normalised first to their respective loading control values and then to their respective CHX untreated control. Data was graphed in Prism and a one-phase decay analysis was performed. Half-life ($t_{1/2}$) values for DR5 are indicated on the graph. Rate constant (k) values for DR5 in SC and siRALB treated cells are 0.0073 and 0.0066 min^{-1}, respectively.

Supplementary Figure 6

Supplementary Figure 6. siRALB sensitises *RAS*MT cells to rhTRAIL-mediated apoptosis. A. CRC cells were transfected with 10nM siRNA targeted against SC or siRALB for 24h (SW403, NCIH498, HDC8) or 48h (LS174T), followed by incubation with rhTRAIL for the indicated time. 'CT' represents samples not treated with rhTRAIL. **Upper.** WB was used to analyse the expression of the indicated proteins. **Lower.** Caspase-3/7 activity was determined on cell lysates from western blots. **B.** Cells were transfected with 10nM of SC or 2 additional siRNA sequences (sequence _4 and _7) against RALB and treated with rhTRAIL. Apoptosis was determined by WB for PARP and cleaved Caspases-8 and 3. **C**. SW620 and GP5d cells were pre-treated with Dinaciclib for 24h, followed by incubation with rhTRAIL for an additional 24h. Apoptosis was assessed by WB for PARP and cleaved caspase-8 and -3 (**left**) and measurement of caspase-3/7 activity (**right**). **D.** HCT116 cells were treated with 10ng/ml TRAIL for 5h. Whole cell lysates were incubated with AMG 655-conjugated dynabeads overnight. DR5-bound beads were magnetically isolated and washed. WB was used to evaluate the expression of the indicated proteins in the immuno-precipitated DR5-bound and input fractions. **E.** HCT116 cells were transfected with 10nM SC or siRALB for 24h and $pTBK1^{Ser172}$, TBK1, PARP, DR5 and RALB levels were determined by WB.

Supplementary Table 1

Supplementary Table 1. Overview of antibodies with supplier, product codes and application used in the manuscript.
